# Explicit Lump Solitary Wave of Certain Interesting (3+1)-Dimensional Waves in Physics via Some Recent Traveling Wave Methods

**DOI:** 10.3390/e21040397

**Published:** 2019-04-15

**Authors:** Mostafa M. A. Khater, Raghda A. M. Attia, Dianchen Lu

**Affiliations:** 1 Department of Mathematics, Faculty of Science, Jiangsu University, Zhenjiang 212013, China; 2 Department of Basic Science, Higher Technological Institute 10th of Ramadan City, El Sharqia 44634, Egypt

**Keywords:** fractional Jimbo–Miwa (JM) equation, explicit lump solitary wave solutions, complex lump solitary wave solutions

## Abstract

This study investigates the solitary wave solutions of the nonlinear fractional Jimbo–Miwa (JM) equation by using the conformable fractional derivative and some other distinct analytical techniques. The JM equation describes the certain interesting (3+1)-dimensional waves in physics. Moreover, it is considered as a second equation of the famous Painlev’e hierarchy of integrable systems. The fractional conformable derivatives properties were employed to convert it into an ordinary differential equation with an integer order to obtain many novel exact solutions of this model. The conformable fractional derivative is equivalent to the ordinary derivative for the functions that has continuous derivatives up to some desired order over some domain (smooth functions). The obtained solutions for each technique were characterized and compared to illustrate the similarities and differences between them. Profound solutions were concluded to be powerful, easy and effective on the nonlinear partial differential equation.

## 1. Introduction

During the last five decades, the nonlinear fractional partial differential equations (NLFPD) have been used for modeling many of the nonlinear phenomena in various fields. For instance, physics, chaos synchronization, continuous-time random walk, mechanical engineering, dynamical and sub-diffusive systems, anomalous diffusive, wave propagation phenomenon and so on. The non-local property is considered as the fundamental advantage of discovering many distinct properties of fractal models. Fractional calculus is a generalization of ordinary calculus, where derivatives and integrals of arbitrary real or complex order are defined. These fractional operators may model more efficiently certain real-world phenomena, especially when the dynamics are affected by constraints inherent to the system. According to the fundamental role of fractal models, an extensive study is applied on the fractional calculus to discover new fractional derivatives that have been defined such as the Riemann–Liouville, Caputo, Hadamard, Riesz, Grünwald–Letnikov, Marchaud, etc. [1,2,3,4,5]. These kinds of derivatives are linear operators and possess some fine properties, but they are not available for all other operational behaviors of a typical first derivative, such as chain rule, quotient rule, semigroup property, and product rule. Failure of previously mentioned derivatives created the interest of many researchers to derive a new derivative until, in 2014, R. Khalil et al. were discovered a new kind of derivative that is a new local fractional derivative which is well-behaved and complies with the computational relationships of the first derivative, called conformable derivative [6,7,8,9,10]. In the following steps, we give the summarized properties of this kind of derivative as follows:

For the given function f:[0,∞[→ℜ, then the conformable derivatives of it with order α where α∈]0,1[, is given by

(1) $$\begin{array}{c} D_{\alpha }\left(f\right)\left(t\right)=\lim_{\varepsilon \to 0}\frac{f\left(t+\varepsilon \;t^{1-\alpha }\right)-f\left(t\right)}{\varepsilon } \end{array}$$

for all t>0. If *f* is α-differentiable in (0,a), a>0, and $\lim_{t\to 0^{+}}f^{\alpha }\left(t\right)$ exists, then define

(2) $$\begin{array}{c} f^{\left(\alpha \right)}\left(0\right)=D_{\alpha }\left(f\right)\left(t\right)=\lim_{t\to 0^{+}}f^{\alpha }\left(t\right). \end{array}$$

For classical definition of R−L and Caputo on polynomial, we see the conformable derivative coincides with them such as it takes the following definition

(3) $$\begin{array}{c} D_{\alpha }\left(t^{m}\right)=m\;t^{m-\alpha }, \end{array}$$

where *m* is arbitrary constant.

For chain rule, quotient rule and semi group property, we see the properties of conformable derivative given by

(4) $$\begin{array}{c} D_{\alpha }\left(a\;f+b\;g\right)=a\;D_{\alpha }\left(f\right)+b\;D_{\alpha }\left(g\right), \end{array}$$

where *a* and *b* are arbitrary constants.

(5) $$\begin{array}{c} D_{\alpha }\left(\lambda \right)=0, \end{array}$$

where λ is arbitrary constant.

(6) $$\begin{array}{c} D_{\alpha }\left(f\;g\right)=f\;D_{\alpha }\left(g\right)+g\;D_{\alpha }\left(f\right), \end{array}$$

(7) $$\begin{array}{c} D_{\alpha }\left(\frac{f}{g}=\frac{g\;D_{\alpha }\left(f\right)-f\;D_{\alpha }\left(g\right)}{g^{2}}\right) \end{array}$$

and also

(8) $$\begin{array}{c} D_{\alpha }\left(f\right)\left(t\right)=t^{1-\alpha }\;\frac{d\;f}{d\;t}\left(t\right) \end{array}$$

when *f* is differentiable. According to these properties and definitions of conformable fractional derivative, it is equivalent to the ordinary derivative for smooth functions [11,12].

**Definition** **1.** *The local fractional derivatives:*

let k:[a,b]→ℜ be a continuous nonnegative map such that k(t)≠0, whenever t>a. Given a function f:[a,b]→ℜ and α∈(0,1) a real, we say that *f* is α-differentiable at t>a, with respect to kernel *k*, if the limit that given by Equation (1), exists. The α derivative at t=a is defined by

(9) $$\begin{array}{c} f^{\left(\alpha \right)}\left(t\right)=\lim_{t\to a^{+}}f^{\left(\alpha \right)}\left(t\right) \end{array}$$

if the limit exists.

Consider the limit $\alpha \to 1^{-}.$ In this case, for t>a, we obtain the classical definition of the fraction that is matched with conformable fractional derivative definition. Based on the conformable derivative definition, the fractional models convert to nonlinear ordinary differential equations with integer order. In this step, the contribution of both analytical and numerical schemes have started to study explicit solutions for these models and for this purpose, many analytical and approximate schemes have been derived such as exp (−ϕ(Θ))-expansion, improved F-expansion, extended $\left(\frac{G^{\prime }}{G}\right)$-expansion, extended tanh- function, simplest and extended simplest equation, generalized Riccati expansion and sinh-Gordon expansion, Riccati–Bernoulli Sub-ODE, and modified Khater methods to discover more physical and dynamical properties of these models. For further information about these methods, you can see [13,14,15,16,17,18,19,20,21,22,23,24,25,26,27].

This research applies a nine recent methods to the nonlinear time fractional Jimbo–Miwa (JM) equation for studying the properties of exact and solitary wave solutions. Moreover, we study the performance of a novel technique that has called the modified auxiliary equation method (modified Khater method) on the same model for examining the similarities and differences between these methods.

In 1983, Jimbo and Miwa put the structure of the JM model in the following formula [28]:

(10) $$\begin{array}{c} \upsilon_{x\;x\;x\;y}+3\;\upsilon_{x\;y}\;\upsilon_{x}+3\;\upsilon_{y}\;\upsilon_{x\;x}+2\;\upsilon_{y\;t}-3\;\upsilon_{x\;z}=0, \end{array}$$

where υ=υ(x,y,z,t) describes a certain wave in physics. This model is the second equation in the well-known KP hierarchy of integrable systems that are given by [29]

(11) $$\begin{array}{c} {\left(u_{t}+6\;u\;u_{x}+u_{x\;x\;x}\right)}_{x}+3\;\sigma^{2}\;u_{y\;y}=0, \end{array}$$

where u=u(x,y,t) is a scalar function, x,y,t,σ represent longitudinal, transverse spatial coordinates, time, and arbitrary constant, respectively. However, it does not pass any of the conventional integrability tests. It is shown to be conditionally integrable having two types of solitary wave solutions such that the solitary waves in second type do not interact elastically. Equation (11) has two forms that depend on the values of σ as follows [30,31,32,33,34]:

- When $\sigma =i,i=\sqrt{-1}$, Equation (11) has been called a KPI equation and was used to describe waves in thin films with high surface tension [35].
- When σ=1, Equation (11) has been named KPII equation and was used to describe water waves with small surface tension [36].

**Figure 1:**
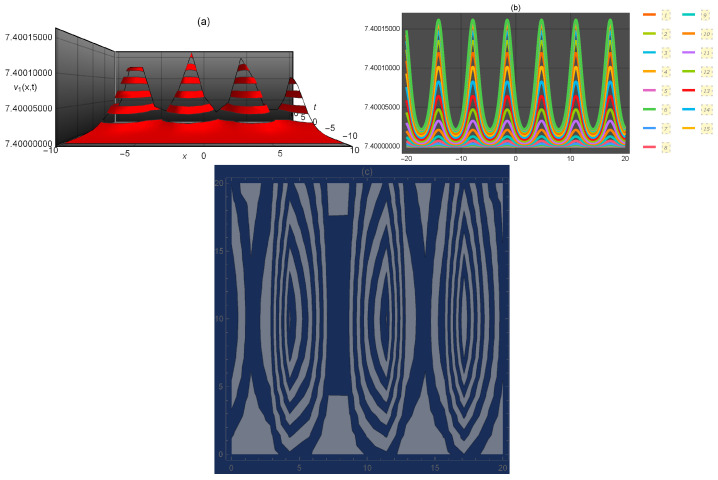
Representation of the solution of Equation (15): (**a**) three-dimensional $\nu_{1}\left(x,t\right)$; (**b**) $\nu_{1}\left(x\right)$ for several values of *t* and (**c**) density plot $\nu_{1}\left(x,t\right)$.

**Figure 2:**
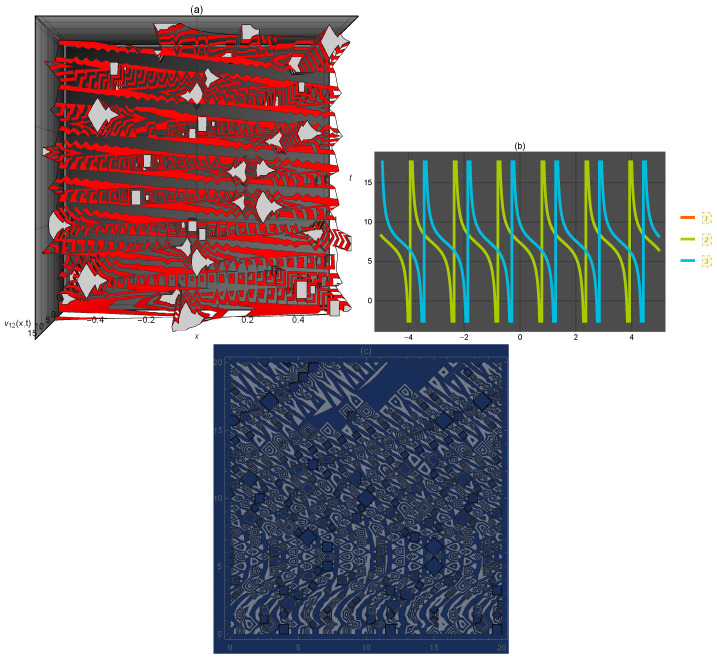
Representation of the solution of Equation (27): (**a**) three-dimensional $\nu_{12}\left(x,t\right)$; (**b**) $\nu_{12}\left(x\right)$ for several values of *t* and (**c**) density plot $\nu_{12}\left(x,t\right)$.

**Figure 3:**
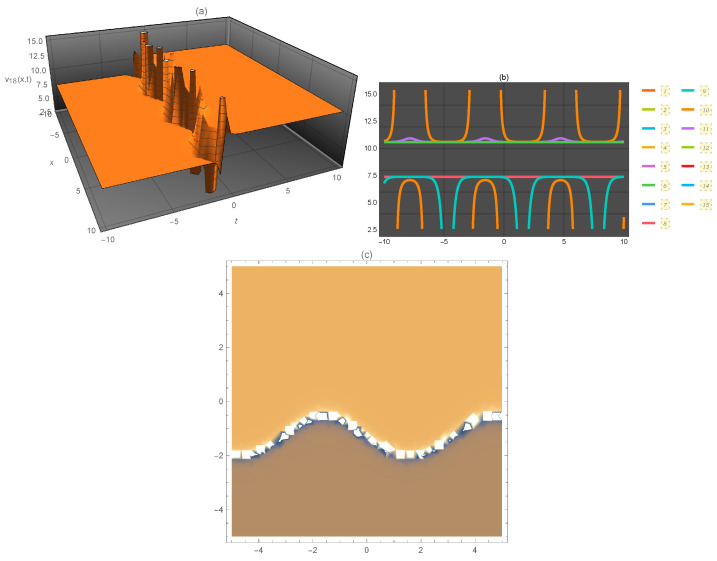
Representation of the solution of Equation (35): (**a**) three-dimensional $\nu_{18}\left(x,t\right)$; (**b**) $\nu_{18}\left(x\right)$ for several values of *t* and (**c**) density plot $\nu_{18}\left(x,t\right)$.

**Figure 4:**
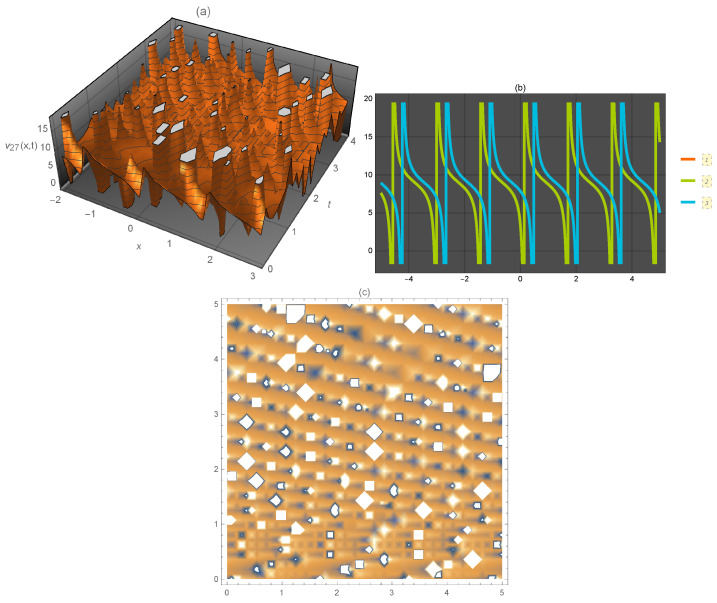
Representation of the solution of Equation (45): (**a**) three-dimensional $\nu_{27}\left(x,t\right)$; (**b**) $\nu_{27}\left(x\right)$ for several values of *t* and (**c**) density plot $\nu_{27}\left(x,t\right)$.

**Figure 5:**
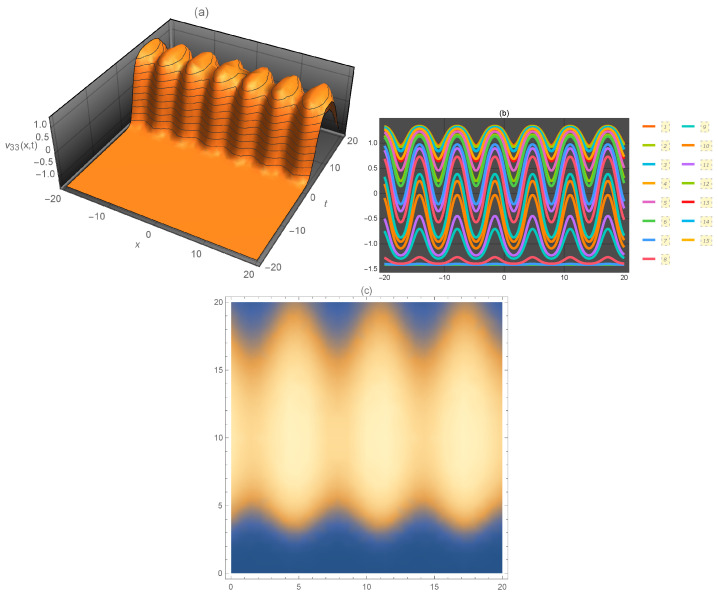
Representation of the solution of Equation (52): (**a**) three-dimensional $\nu_{33}\left(x,t\right)$; (**b**) $\nu_{33}\left(x\right)$ for several values of *t* and (**c**) density plot $\nu_{33}\left(x,t\right)$.

**Figure 6:**
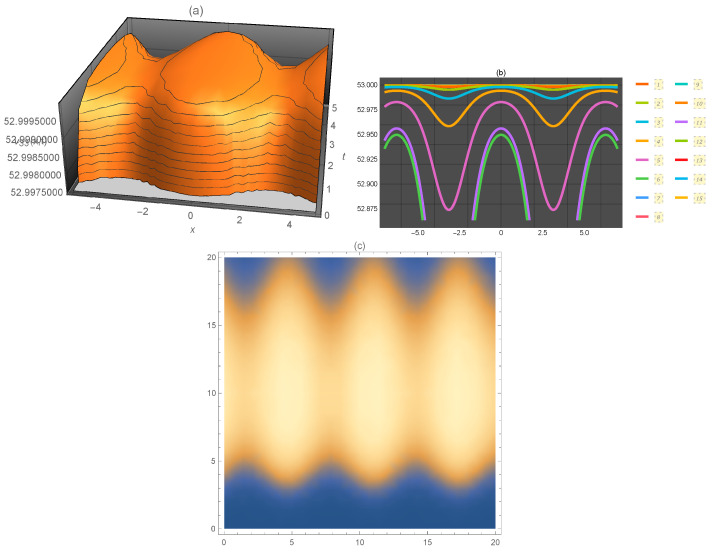
Representation of the solution of Equation (58): (**a**) three-dimensional $\nu_{38}\left(x,t\right)$; (**b**) $\nu_{38}\left(x\right)$ for several values of *t* and (**c**) density plot $\nu_{38}\left(x,t\right)$.

**Figure 7:**
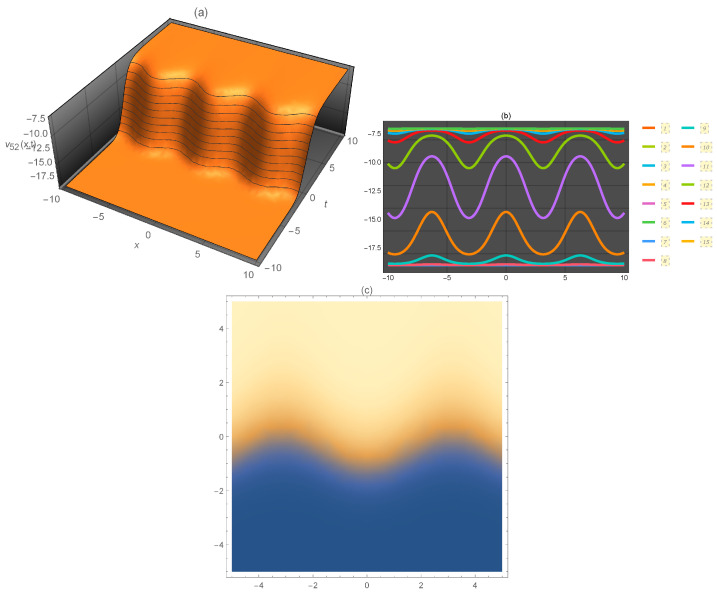
Representation of the solution of Equation (73): (**a**) three-dimensional $\nu_{52}\left(x,t\right)$; (**b**) $\nu_{52}\left(x\right)$ for several values of *t* and (**c**) density plot $\nu_{52}\left(x,t\right)$.

**Figure 8:**
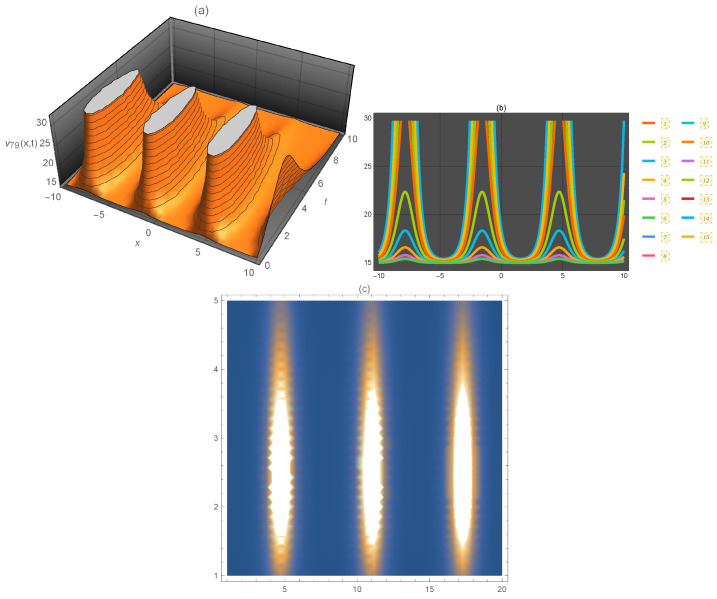
Representation of the solution of Equation (101): (**a**) three-dimensional $\nu_{79}\left(x,t\right)$; (**b**) $\nu_{79}\left(x\right)$ for several values of *t* and (**c**) density plot $\nu_{79}\left(x,t\right)$.

**Figure 9:**
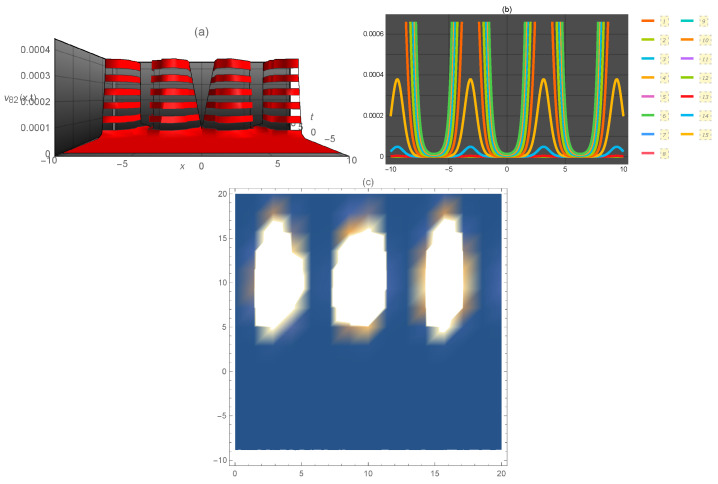
Representation of the solution of Equation (105): (**a**) three-dimensional $\nu_{82}\left(x,t\right)$; (**b**) $\nu_{82}\left(x\right)$ for several values of *t* and (**c**) density plot $\nu_{82}\left(x,t\right)$.

**Figure 10:**
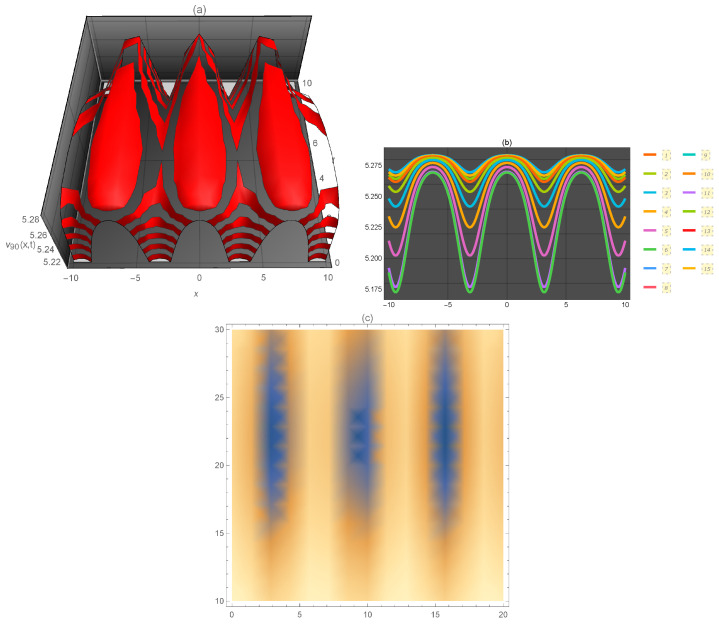
Representation of the solution of Equation (114): (**a**) three-dimensional $\nu_{90}\left(x,t\right)$; (**b**) $\nu_{90}\left(x\right)$ for several values of *t* and (**c**) density plot $\nu_{90}\left(x,t\right)$.

The rest of the paper is organized as follows: Section 2 applies the above-mentioned techniques to the nonlinear time fractional JM equation to obtain exact and solitary wave solutions. Section 3 sketches some figures (Figure 1, Figure 2, Figure 3, Figure 4, Figure 5, Figure 6, Figure 7, Figure 8, Figure 9 and Figure 10) for the solutions and gives the physical interpretation of them. Section 4 gives a full discussion about the similarities and differences between these methods. Section 5 summaries the main conclusions.

## 2. Explicit Wave Solutions of the Nonlinear Time Fractional JM Model

This part tests the performance of ten analytical techniques on the nonlinear time fractional Jimbo–Miwa (JM). This model has a variety of solutions with distinct structures such as single-soliton solutions, multiple-soliton solutions, periodic wave solutions, and traveling wave solutions [37,38,39,40,41,42,43,44]. The fractional mathematical modeling of this equation is given as follows:

(12) $$\begin{array}{c} \nu_{xxxy}+p\;\nu_{y}\;\nu_{xx}+q\;\nu_{x}\;\nu_{xy}+r\;D_{t}^{\alpha_{1}}\nu_{y}-s\;\nu_{xz}=0, \end{array}$$

where ν=ν(x,y,z,t) describes the dynamics of a certain (3+1)-dimensional waves in physics and p,q,r,s are arbitrary constants. Transformation the NLFPD equation into the NLPD equation with integer order by using the following conformable fractional derivative $\left[\nu \left(x,t\right)=\nu \left(\Theta \right),\;\Theta =x+y+z+\frac{c\;t^{\alpha_{1}}}{\alpha_{1}}\right]$ yields:

(13) $$\begin{array}{c} \nu^{\prime \prime \prime }+\frac{1}{2}\;\left(p+q\right)\;{\nu^{\prime }}^{2}+\left(r\;c-s\right)\;u^{\prime }=0. \end{array}$$

Calculating the homogeneous balance value between the highest order derivative and nonlinear terms of Equation (13) gives N=1.

### 2.1. Utilization of Exp (−ϕ(Θ))-Expansion Method

Applying this method enables putting the general solution of Equation (13) in the next formula:

(14) $$\begin{array}{c} \nu \left(\Theta \right)=\sum_{i=0}^{N}a_{i}e^{-i\phi (\Theta )}=a_{1}\;e^{-\phi (\Theta )}+a_{0}, \end{array}$$

where $a_{i},\left\{i=0,1,\cdots ,N\right\}$ is arbitrary constant and ϕ(Θ) is the solution function of the next ODE

$$\begin{array}{c} \phi^{\prime }\left(\Theta \right)=\lambda +\mu e^{\phi (\Theta )}+\frac{1}{e^{\phi (\Theta )}}, \end{array}$$

where λ, and μ are arbitrary constants. Handling Equation (13) by utilizing Equation (14) and its derivatives, converts the left-hand side of Equation (13) to a polynomial equation of $e^{\left(-\phi (\Theta )\right)}$. Gathering all coefficients of term that has the same degree and equating them to zero. Solving the obtained algebraic system of equations leads to

$$a_{1}\to \frac{12}{p+q},c\to -\frac{\lambda^{2}-4\mu -s}{r},\;\mathrm{where}\;\;\left[p+q\neq 0,\;\lambda^{2}-4\mu \neq s,\;r\neq 0\right].$$

According to the value of these parameters, we get the relevant traveling wave solutions of Equation (12):

When $\left[\lambda^{2}-4\;\mu >0,\;\mu \neq 0\right]$:

(15) $$\begin{array}{c} \nu_{1}\left(x,y,z,t\right)=a_{0}-\frac{24\mu }{\left(p+q\right)(\lambda -\sqrt{\lambda^{2}-4\mu }\tanh\left(\frac{1}{2}\sqrt{\lambda^{2}-4\mu }\left(\frac{t^{\alpha_{1}}\left(-\lambda^{2}+4\mu +s\right)}{\alpha_{1}r}+x+y+z+\vartheta \right)\right))}, \end{array}$$

(16) $$\begin{array}{c} \nu_{2}\left(x,y,z,t\right)=a_{0}-\frac{24\mu }{\left(p+q\right)(\lambda -\sqrt{\lambda^{2}-4\mu }\coth\left(\frac{1}{2}\sqrt{\lambda^{2}-4\mu }\left(\frac{t^{\alpha_{1}}\left(-\lambda^{2}+4\mu +s\right)}{\alpha_{1}r}+x+y+z+\vartheta \right)\right))}. \end{array}$$

When $\left[\lambda^{2}-4\;\mu >0,\;\mu =0\right]$:

(17) $$\begin{array}{c} \nu_{3}\left(x,y,z,t\right)=a_{0}+\frac{12\lambda }{\left(p+q\right)(\exp\left(\lambda \left(\frac{t^{\alpha_{1}}\left(-\lambda^{2}+s\right)}{\alpha_{1}r}+x+y+z+\vartheta \right)\right)-1)}. \end{array}$$

When $\left[\lambda^{2}-4\;\mu =0,\;\mu \neq 0,\;\lambda \neq 0\right]$:

(18) $$\begin{array}{c} \nu_{4}\left(x,y,z,t\right)=a_{0}-\frac{6\lambda^{2}\left(\alpha_{1}r\left(x+y+z+\vartheta \right)+s\;t^{\alpha_{1}}\right)}{\left(p+q\right)(\alpha_{1}r\left(\lambda \left(x+y+z+\vartheta \right)+2\right)+\lambda \;s\;t^{\alpha_{1}})}. \end{array}$$

When $\left[\lambda^{2}-4\;\mu =0,\;\mu =0,\;\lambda =0\right]$:

(19) $$\begin{array}{c} \nu_{5}\left(x,y,z,t\right)=a_{0}+\frac{12\alpha_{1}r}{\left(p+q\right)(\alpha_{1}r\left(x+y+z+\vartheta \right)+s\;t^{\alpha_{1}})}. \end{array}$$

When $\left[\lambda^{2}-4\;\mu <0,\;\mu \neq 0\right]$:

(20) $$\begin{array}{c} \nu_{6}\left(x,y,z,t\right)=a_{0}-\frac{24\mu }{\left(p+q\right)(\lambda -\sqrt{4\mu -\lambda^{2}}\tan\left(\frac{1}{2}\sqrt{4\mu -\lambda^{2}}\left(\frac{t^{\alpha_{1}}\left(-\lambda^{2}+4\mu +s\right)}{\alpha_{1}r}+x+y+z+\vartheta \right)\right))}, \end{array}$$

(21) $$\begin{array}{c} \nu_{7}\left(x,y,z,t\right)=a_{0}-\frac{24\mu }{\left(p+q\right)(\lambda -\sqrt{4\mu -\lambda^{2}}\cot\left(\frac{1}{2}\sqrt{4\mu -\lambda^{2}}\left(\frac{t^{\alpha_{1}}\left(-\lambda^{2}+4\mu +s\right)}{\alpha_{1}r}+x+y+z+\vartheta \right)\right))}. \end{array}$$

### 2.2. Utilization of the Improved F-Expansion Method

Applying this method enables putting the general solution of Equation (13) in the next formula:

(22) $$\begin{array}{c} \nu \left(\Theta \right)=\sum_{i=-N}^{N}a_{i}{\left(\mu +\phi \left(\Theta \right)\right)}^{i}=\frac{a_{-1}}{\mu +\phi (\Theta )}+a_{1}\left(\mu +\phi \left(\Theta \right)\right)+a_{0}, \end{array}$$

where $a_{i},\;\left\{i=-N,-N+1,\cdots ,\;N-1,N\right\}$ is arbitrary constant and ϕ(Θ) is the solution of the next ODE

$$\begin{array}{c} \phi^{\prime }\left(\Theta \right)=\phi {\left(\Theta \right)}^{2}+r. \end{array}$$

Handling of Equation (13) by utilizing Equation (22) and its derivatives converts the left-hand side of Equation (13) to a polynomial function of ϕ(Θ). Gather all coefficients of terms that have the same degree and equate them to zero. Solving the obtained system of equations yields.

*Family I*:

$$a_{-1}\to \frac{12(\mu^{2}+r)}{p+q},a_{1}\to 0,c\to \frac{4r+s}{r},\;\mathrm{where}\;\;\left[4\;r+s\neq 0,\;r\neq 0,\;p+q\neq =0\right].$$

According to the value of these parameters, we get the relevant traveling wave solutions of Equation (12):

When [r<0]:

(23) $$\begin{array}{c} \nu_{8}\left(x,y,z,t\right)=a_{0}+\frac{12(\mu^{2}+r)}{\left(p+q\right)(\mu +\sqrt{r}\tan\left(\sqrt{r}\left(\frac{\left(4r+s\right)t^{\alpha_{1}}}{\alpha_{1}r}+x+y+z\right)\right))}, \end{array}$$

(24) $$\begin{array}{c} \nu_{9}\left(x,y,z,t\right)=a_{0}+\frac{12(\mu^{2}+r)}{\left(p+q\right)(\mu -\sqrt{r}\cot\left(\sqrt{r}\left(\frac{\left(4r+s\right)t^{\alpha_{1}}}{\alpha_{1}r}+x+y+z\right)\right))}. \end{array}$$

When [r>0]:

(25) $$\begin{array}{c} \nu_{10}\left(x,y,z,t\right)=a_{0}+\frac{12(\mu^{2}+r)}{\left(p+q\right)(\mu +\sqrt{r}\tan\left(\sqrt{r}\left(\frac{\left(4r+s\right)t^{\alpha_{1}}}{\alpha_{1}r}+x+y+z\right)\right))}, \end{array}$$

(26) $$\begin{array}{c} \nu_{11}\left(x,y,z,t\right)=a_{0}+\frac{12(\mu^{2}+r)}{\left(p+q\right)(\mu -\sqrt{r}\cot\left(\sqrt{r}\left(\frac{\left(4r+s\right)t^{\alpha_{1}}}{\alpha_{1}r}+x+y+z\right)\right))}. \end{array}$$

*Family II*:

$$a_{-1}\to 0,a_{1}\to -\frac{12}{p+q},c\to \frac{4r+s}{r},\;\mathrm{where}\;\;\left[4\;r+s\neq 0,\;r\neq 0,\;p+q\neq =0\right].$$

According to the value of these parameters, we get the relevant traveling wave solutions of Equation (12):

When [r<0]:

(27) $$\begin{array}{c} \nu_{12}\left(x,y,z,t\right)=\frac{a_{0}\left(p+q\right)-12\left(\mu +\sqrt{r}\tan\left(\sqrt{r}\left(\frac{\left(4r+s\right)t^{\alpha_{1}}}{\alpha_{1}r}+x+y+z\right)\right)\right)}{p+q}, \end{array}$$

(28) $$\begin{array}{c} \nu_{13}\left(x,y,z,t\right)=a_{0}-\frac{12(\mu -\sqrt{r}\cot\left(\sqrt{r}\left(\frac{\left(4r+s\right)t^{\alpha_{1}}}{\alpha_{1}r}+x+y+z\right)\right))}{p+q}. \end{array}$$

When [r>0]:

(29) $$\begin{array}{c} \nu_{14}\left(x,y,z,t\right)=a_{0}-\frac{12(\mu +\sqrt{r}\tan\left(\sqrt{r}\left(\frac{\left(4r+s\right)t^{\alpha_{1}}}{\alpha_{1}r}+x+y+z\right)\right))}{p+q}, \end{array}$$

(30) $$\begin{array}{c} \nu_{15}\left(x,y,z,t\right)=a_{0}-\frac{12(\mu -\sqrt{r}\cot\left(\sqrt{r}\left(\frac{\left(4r+s\right)t^{\alpha_{1}}}{\alpha_{1}r}+x+y+z\right)\right))}{p+q}. \end{array}$$

### 2.3. Utilization of an Extended $\left(\frac{G^{\prime }}{G}\right)$-Expansion Method

Applying this method enables putting the general solution of Equation (13) in the next formula:

(31) $$\begin{array}{cc} \nu \left(\Theta \right)=a_{0}+\sum_{i=1}^{N} & \left(a_{i}\;{\left(\frac{G^{\prime }\left(\Theta \right)}{G(\Theta )}\right)}^{i}+b_{i}{\left(\frac{G^{\prime }\left(\Theta \right)}{G(\Theta )}\right)}^{i-1}\sqrt{\sigma \left(\frac{{\left(\frac{G^{\prime }\left(\Theta \right)}{G(\Theta )}\right)}^{2}}{\mu }+1\right)}\right)+a_{0}\\ & =\frac{a_{1}G^{\prime }\left(\Theta \right)}{G(\Theta )}+a_{0}+b_{1}\sqrt{\sigma (\frac{G^{\prime }{\left(\Theta \right)}^{2}}{\mu G{\left(\Theta \right)}^{2}}+1)}, \end{array}$$

where $a_{i},b_{i}$ are arbitrary constants and $\frac{G^{\prime }\left(\Theta \right)}{G(\Theta )}$ is the solutions of the next ODE

$$\begin{array}{c} \frac{G^{\prime \prime }\left(\Theta \right)}{G(\Theta )}=-\mu , \end{array}$$

where μ is arbitrary constant. Handling of Equation (13) by utilizing Equation (31) and its derivatives converts the left-hand side of Equation (13) to the polynomial function of ${\left(\frac{G^{\prime }\left(\Theta \right)}{G(\Theta )}\right)}^{i}{\left[\sqrt{\frac{\sigma G^{\prime }{\left(\Theta \right)}^{2}}{\mu G{\left(\Theta \right)}^{2}}+\sigma }\right]}^{j},$ where {i=0,1,⋯,4&j=0,1}. Gather all coefficients of terms that have the same degree and equate them to zero. Solving the obtained system of equation yields

*Family I:*

$$a_{1}\to \frac{6}{p+q},b_{1}\to \frac{6\sqrt{\mu }}{\sqrt{p^{2}\sigma +2pq\sigma +q^{2}\sigma }},c\to \frac{\mu +s}{r},\;\mathrm{where}\;\;\left[p^{2}\sigma +2pq\sigma +q^{2}\sigma >0,\;p+q\neq 0,\;r\neq 0\right],$$

$$\left[\mu >0,\;\mu +s\neq 0\right].$$

According to the value of these parameters, the relevant traveling wave solutions of Equation (12) are given by the following formulas:

When [μ>0]:

(32) $$\begin{array}{cc} \nu_{16}\left(x,y,z,t\right)= & a_{0}+\frac{6\sqrt{\mu }\sqrt{\frac{(C_{1}^{2}+C_{2}^{2})\sigma }{\left(C_{1}\sin\left(\sqrt{\mu }\left(\frac{\left(\mu +s\right)t^{\alpha_{1}}}{\alpha_{1}r}+x+y+z\right)\right)+C_{2}\cos\left(\sqrt{\mu }\left(\frac{\left(\mu +s\right)t^{\alpha_{1}}}{\alpha_{1}r}+x+y+z\right)\right)\right){}^{2}}}}{\sqrt{\sigma {\left(p+q\right)}^{2}}}\\ & \;+\frac{6\sqrt{\mu }\left(C_{1}-C_{2}\tan\left(\sqrt{\mu }\left(\frac{\left(\mu +s\right)t^{\alpha_{1}}}{\alpha_{1}r}+x+y+z\right)\right)\right)}{\left(p+q\right)(C_{1}\tan\left(\sqrt{\mu }\left(\frac{\left(\mu +s\right)t^{\alpha_{1}}}{\alpha_{1}r}+x+y+z\right)\right)+C_{2})}. \end{array}$$

When [μ<0]:

(33) $$\begin{array}{c} \nu_{17}\left(x,y,z,t\right)=a_{0}+\frac{6\sqrt{\mu }\sqrt{\frac{(C_{1}^{2}-C_{2}^{2})\sigma }{\left(C_{1}\cos\left(\sqrt{\mu }\left(\frac{\left(\mu +s\right)t^{\alpha_{1}}}{\alpha_{1}r}+x+y+z\right)\right)+C_{2}\sinh\left(\sqrt{-\mu }\left(\frac{\left(\mu +s\right)t^{\alpha_{1}}}{\alpha_{1}r}+x+y+z\right)\right)\right){}^{2}}}}{\sqrt{\sigma {\left(p+q\right)}^{2}}}\\ \;+\frac{6C_{2}\sqrt{-\mu }\cos\left(\sqrt{\mu }\left(\frac{\left(\mu +s\right)t^{\alpha_{1}}}{\alpha_{1}r}+x+y+z\right)\right)}{\left(p+q\right)(C_{1}\cos\left(\sqrt{\mu }\left(\frac{\left(\mu +s\right)t^{\alpha_{1}}}{\alpha_{1}r}+x+y+z\right)\right)+C_{2}\sinh\left(\sqrt{-\mu }\left(\frac{\left(\mu +s\right)t^{\alpha_{1}}}{\alpha_{1}r}+x+y+z\right)\right))}\\ \;-\frac{6C_{1}\sqrt{\mu }\sin\left(\sqrt{\mu }\left(\frac{\left(\mu +s\right)t^{\alpha_{1}}}{\alpha_{1}r}+x+y+z\right)\right)}{\left(p+q\right)(C_{1}\cos\left(\sqrt{\mu }\left(\frac{\left(\mu +s\right)t^{\alpha_{1}}}{\alpha_{1}r}+x+y+z\right)\right)+C_{2}\sinh\left(\sqrt{-\mu }\left(\frac{\left(\mu +s\right)t^{\alpha_{1}}}{\alpha_{1}r}+x+y+z\right)\right))}. \end{array}$$

*Family II:*

$$a_{1}\to \frac{12}{p+q},b_{1}\to 0,c\to \frac{4\mu +s}{r},\mathrm{where}\;\;\left[p+q\neq 0,\;r\neq 0,\;4\;\mu +s\neq 0\right].$$

According to the value of these parameters, the relevant traveling wave solutions of Equation (12) are given by the following formulas:

When [μ>0]:

(34) $$\begin{array}{c} \nu_{17}\left(x,y,z,t\right)=\frac{\left(a_{0}C_{1}\left(p+q\right)-12C_{2}\sqrt{\mu }\right)\sin\left(\sqrt{\mu }\left(\frac{\left(4\mu +s\right)t^{\alpha_{1}}}{\alpha_{1}r}+x+y+z\right)\right)}{\left(p+q\right)(C_{1}\sin\left(\sqrt{\mu }\left(\frac{\left(4\mu +s\right)t^{\alpha_{1}}}{\alpha_{1}r}+x+y+z\right)\right)+C_{2}\cos\left(\sqrt{\mu }\left(\frac{\left(4\mu +s\right)t^{\alpha_{1}}}{\alpha_{1}r}+x+y+z\right)\right))}\\ +\frac{\left(a_{0}C_{2}\left(p+q\right)+12C_{1}\sqrt{\mu }\right)\cos\left(\sqrt{\mu }\left(\frac{\left(4\mu +s\right)t^{\alpha_{1}}}{\alpha_{1}r}+x+y+z\right)\right)}{\left(p+q\right)(C_{1}\sin\left(\sqrt{\mu }\left(\frac{\left(4\mu +s\right)t^{\alpha_{1}}}{\alpha_{1}r}+x+y+z\right)\right)+C_{2}\cos\left(\sqrt{\mu }\left(\frac{\left(4\mu +s\right)t^{\alpha_{1}}}{\alpha_{1}r}+x+y+z\right)\right))}. \end{array}$$

When [μ<0]:

(35) $$\begin{array}{c} \nu_{18}\left(x,y,z,t\right)=\frac{C_{1}\left(a_{0}\left(p+q\right)\cos\left(\sqrt{\mu }\left(\frac{\left(4\mu +s\right)t^{\alpha_{1}}}{\alpha_{1}r}+x+y+z\right)\right)-12\sqrt{\mu }\sin\left(\sqrt{\mu }\left(\frac{\left(4\mu +s\right)t^{\alpha_{1}}}{\alpha_{1}r}+x+y+z\right)\right)\right)}{\left(p+q\right)(C_{1}\cos\left(\sqrt{\mu }\left(\frac{\left(4\mu +s\right)t^{\alpha_{1}}}{\alpha_{1}r}+x+y+z\right)\right)+C_{2}\sinh\left(\sqrt{-\mu }\left(\frac{\left(4\mu +s\right)t^{\alpha_{1}}}{\alpha_{1}r}+x+y+z\right)\right))}\\ +\frac{C_{2}\left(a_{0}\left(p+q\right)\sinh\left(\sqrt{-\mu }\left(\frac{\left(4\mu +s\right)t^{\alpha_{1}}}{\alpha_{1}r}+x+y+z\right)\right)+12\sqrt{-\mu }\cos\left(\sqrt{\mu }\left(\frac{\left(4\mu +s\right)t^{\alpha_{1}}}{\alpha_{1}r}+x+y+z\right)\right)\right)}{\left(p+q\right)(C_{1}\cos\left(\sqrt{\mu }\left(\frac{\left(4\mu +s\right)t^{\alpha_{1}}}{\alpha_{1}r}+x+y+z\right)\right)+C_{2}\sinh\left(\sqrt{-\mu }\left(\frac{\left(4\mu +s\right)t^{\alpha_{1}}}{\alpha_{1}r}+x+y+z\right)\right))}. \end{array}$$

### 2.4. Utilization of an Extended Tanh-Function Method

Applying this method enables putting the general solution of Equation (13) into the next formula:

(36) $$\begin{array}{c} \nu \left(\Theta \right)=\sum_{i=-N}^{N}a_{i}\phi {\left(\Theta \right)}^{i}=\frac{a_{-1}}{\phi (\Theta )}+a_{1}\phi \left(\Theta \right)+a_{0}, \end{array}$$

where $a_{i},\left\{i=-N,-N+1,\cdots ,N-1,N\right\}$ and ϕ(Θ) is the solution of the next ODE

$$\begin{array}{c} \phi^{\prime }\left(\Theta \right)=d+\phi {\left(\Theta \right)}^{2}, \end{array}$$

where *d* is arbitrary constants. Handling of Equation (13) by utilizing Equation (36) and its derivatives converts the left-hand side of Equation (13) to polynomial function of ϕ(Θ). Gather all coefficients of terms that have a same degree and equate them to zero. Solving the obtained system of equation results in

*Family I:*

$$a_{1}\to -\frac{12}{p+q},a_{-1}\to \frac{12d}{p+q},c\to \frac{16d+s}{r},\mathrm{where}\left[p+q\neq 0,\;r\neq 0,\;16\;d+s\neq 0\right].$$

According to the value of these parameters, the relevant traveling wave solutions of Equation (12) are given by the following formulas:

When [d<0]:

(37) $$\begin{array}{c} \nu_{19}\left(x,y,z,t\right)=a_{0}+\frac{12\sqrt{d}\tan\left(\sqrt{d}\left(\frac{\left(16d+s\right)t^{\alpha_{1}}}{\alpha_{1}r}+x+y+z\right)\right)\left(\cot^{2}\left(\sqrt{d}\left(\frac{\left(16d+s\right)t^{\alpha_{1}}}{\alpha_{1}r}+x+y+z\right)\right)-1\right)}{p+q}. \end{array}$$

When [d>0]:

(38) $$\begin{array}{c} \nu_{20}\left(x,y,z,t\right)=a_{0}+\frac{12\sqrt{d}\tan\left(\sqrt{d}\left(\frac{\left(16d+s\right)t^{\alpha_{1}}}{\alpha_{1}r}+x+y+z\right)\right)\left(\cot^{2}\left(\sqrt{d}\left(\frac{\left(16d+s\right)t^{\alpha_{1}}}{\alpha_{1}r}+x+y+z\right)\right)-1\right)}{p+q}. \end{array}$$

When [d=0]:

(39) $$\begin{array}{c} \nu_{21}\left(x,y,z,t\right)=a_{0}+\frac{12}{\left(p+q\right)\left(\frac{s\;t^{\alpha_{1}}}{\alpha_{1}r}+x+y+z\right)}. \end{array}$$

*Family II:*

$$a_{1}\to -\frac{12}{p+q},a_{-1}\to 0,c\to \frac{4d+s}{r},\mathrm{where}\left[p+q\neq 0,\;r\neq 0,\;4\;d+s\neq 0\right].$$

According to the value of these parameters, the relevant traveling wave solutions of Equation (12) are given by the next formulas:

When [d<0]:

(40) $$\begin{array}{c} \nu_{22}\left(x,y,z,t\right)=a_{0}-\frac{12\sqrt{d}\tan\left(\sqrt{d}\left(\frac{\left(16d+s\right)t^{\alpha_{1}}}{\alpha_{1}r}+x+y+z\right)\right)}{p+q}, \end{array}$$

(41) $$\begin{array}{c} \nu_{23}\left(x,y,z,t\right)=a_{0}+\frac{12\sqrt{d}\cot\left(\sqrt{d}\left(\frac{\left(16d+s\right)t^{\alpha_{1}}}{\alpha_{1}r}+x+y+z\right)\right)}{p+q}. \end{array}$$

When [d>0]:

(42) $$\begin{array}{c} \nu_{24}\left(x,y,z,t\right)=a_{0}-\frac{12\sqrt{d}\tan\left(\sqrt{d}\left(\frac{\left(16d+s\right)t^{\alpha_{1}}}{\alpha_{1}r}+x+y+z\right)\right)}{p+q}, \end{array}$$

(43) $$\begin{array}{c} \nu_{25}\left(x,y,z,t\right)=a_{0}+\frac{12\sqrt{d}\cot\left(\sqrt{d}\left(\frac{\left(16d+s\right)t^{\alpha_{1}}}{\alpha_{1}r}+x+y+z\right)\right)}{p+q}. \end{array}$$

When [d=0]:

(44) $$\begin{array}{c} \nu_{26}\left(x,y,z,t\right)=a_{0}+\frac{12}{\left(p+q\right)\left(\frac{st^{\alpha_{1}}}{\alpha_{1}r}+x+y+z\right)}. \end{array}$$

*Family III:*

$$a_{1}\to 0,a_{-1}\to \frac{12d}{p+q},c\to \frac{4d+s}{r},\mathrm{where}\left[p+q\neq 0,\;r\neq 0,\;4\;d+s\neq 0,\;d\neq 0\right].$$

According to the value of these parameters, the relevant traveling wave solutions of Equation (12) are given by the following formulas:

When [d<0]:

(45) $$\begin{array}{c} \nu_{27}\left(x,y,z,t\right)=a_{0}+\frac{12\sqrt{d}\cot\left(\sqrt{d}\left(\frac{\left(16d+s\right)t^{\alpha_{1}}}{\alpha_{1}r}+x+y+z\right)\right)}{p+q}, \end{array}$$

(46) $$\begin{array}{c} \nu_{28}\left(x,y,z,t\right)=a_{0}-\frac{12\sqrt{d}\tan\left(\sqrt{d}\left(\frac{\left(16d+s\right)t^{\alpha_{1}}}{\alpha_{1}r}+x+y+z\right)\right)}{p+q}. \end{array}$$

When [d>0]:

(47) $$\begin{array}{c} \nu_{29}\left(x,y,z,t\right)=a_{0}+\frac{12\sqrt{d}\cot\left(\sqrt{d}\left(\frac{\left(16d+s\right)t^{\alpha_{1}}}{\alpha_{1}r}+x+y+z\right)\right)}{p+q}, \end{array}$$

(48) $$\begin{array}{c} \nu_{30}\left(x,y,z,t\right)=a_{0}-\frac{12\sqrt{d}\tan\left(\sqrt{d}\left(\frac{\left(16d+s\right)t^{\alpha_{1}}}{\alpha_{1}r}+x+y+z\right)\right)}{p+q}. \end{array}$$

### 2.5. Utilization of the Simplest Equation Method

Applying this method enables putting the general solution of Equation (13) in the next formula:

(49) $$\begin{array}{c} \nu \left(\Theta \right)=\sum_{i=0}^{N}a_{i}f{\left(\Theta \right)}^{i}=a_{1}f\left(\Theta \right)+a_{0}, \end{array}$$

where $a_{i}$ is arbitrary constant and f(Θ) is the solutions of the next ODE

$$\begin{array}{c} f^{\prime }\left(\Theta \right)=c_{2}f{\left(\Theta \right)}^{2}+c_{1}f\left(\Theta \right), \end{array}$$

where $c_{1}\mathrm{and}c_{2}$ are arbitrary constants. Handling of Equation (13) by utilizing Equation (49) and its derivatives converts the left-hand side of Equation (13) to polynomial function of f(Θ). Gathering all coefficients of terms that have the same degree and equating them to zero. Solving the obtained system of equation obtains

*Family I*

$$a_{1}\to -\frac{i\sqrt{2}c}{\sqrt{b}},a\to a_{0}^{2}\left(-b\right),c_{1}\to -\frac{i\sqrt{2}a_{0}\sqrt{b}}{c},\mathrm{where}\;\left[b<0,\;c\neq 0\right].$$

According to the value of these parameters, the relevant traveling wave solutions of Equation (12) are given as follows:

When $\left[c_{2}\to -1\right]$:

(50) $$\begin{array}{c} \nu_{31}\left(x,y,z,t\right)=\sqrt{-\frac{a}{b}}-\frac{icc_{1}\left(1+\tanh\left(\frac{1}{2}c_{1}\left(-\frac{i\sqrt{2}\sqrt{b}\sqrt{-\frac{a}{b}}t^{\alpha_{1}}}{\alpha_{1}c_{1}}+x+y+z+\vartheta \right)\right)\right)}{\sqrt{2}\sqrt{b}}, \end{array}$$

(51) $$\begin{array}{c} \nu_{32}\left(x,y,z,t\right)=\sqrt{-\frac{a}{b}}+\frac{icc_{1}\left(-1+\tanh\left(\frac{1}{2}c_{1}\left(-\frac{i\sqrt{2}\sqrt{b}\sqrt{-\frac{a}{b}}t^{\alpha_{1}}}{\alpha_{1}c_{1}}+x+y+z+\vartheta \right)\right)\right)}{\sqrt{2}\sqrt{b}}. \end{array}$$

*Family II*

$$a_{1}\to -2a_{0},c\to i\sqrt{2}a_{0}\sqrt{b},a\to a_{0}^{2}\left(-b\right),\mathrm{where}\;\left[b<0\right].$$

According to the value of these parameters, the relevant traveling wave solutions of Equation (12) are given as follows:

When $\left[c_{1}\to 1,c_{2}\to -1\right]$:

(52) $$\begin{array}{c} \nu_{33}\left(x,y,z,t\right)=-\sqrt{-\frac{a}{b}}\tanh\left(\frac{1}{2}\left(\frac{\sqrt{2}i\sqrt{b}\sqrt{-\frac{a}{b}}t^{\alpha_{1}}}{\alpha_{1}}+x+y+z\right)\right). \end{array}$$

### 2.6. Utilization of an Extended Simplest Equation Method

Applying this method enables putting the general solution of Equation (13) in the next formula:

(53) $$\begin{array}{c} \nu \left(\Theta \right)=\sum_{i=-N}^{N}a_{i}f{\left(\xi \right)}^{i}=\frac{a_{-1}}{f(\Theta )}+a_{1}f\left(\Theta \right)+a_{0}, \end{array}$$

where $a_{i},\;\left\{i=-N,-N+1,\cdots ,N-1,N\right\}$ and f(Θ) is the solution of the next ODE

$$\begin{array}{c} f^{\prime }\left(\Theta \right)=\alpha +\lambda f\left(\Theta \right)+\mu f{\left(\Theta \right)}^{2}, \end{array}$$

where α,λ,andμ are arbitrary constants. Handling Equation (13) by utilizing Equation (53) and its derivatives converts the left-hand side of Equation (13) to polynomial function of f(Θ). Gather all coefficients of terms that have the same degree and equate them to zero. Solving the obtained system of equation, we get:

*Family I*

$$a_{1}\to -\frac{12\mu }{p+q},a_{-1}\to 0,c\to -\frac{-4\alpha \mu +\lambda^{2}-s}{r},\mathrm{where}\;\left[r\neq 0,\;p+q\neq 0,\;\mu \neq 0,\;-4\;\alpha \;\mu +\lambda^{2}\neq s\right].$$

According to the value of these parameters, the relevant traveling wave solutions of Equation (12) are given as follows:

When [λ=0]:

Thus, when [αμ>0], the solutions take the following forms:

(54) $$\begin{array}{c} \nu_{34}\left(x,y,z,t\right)=a_{0}-\frac{12\sqrt{\alpha \mu }\tan\left(\sqrt{\alpha \mu }\left(\frac{t^{\alpha_{1}}\left(4\alpha \mu +s\right)}{\alpha_{1}r}+x+y+z+\vartheta \right)\right)}{p+q}, \end{array}$$

(55) $$\begin{array}{c} \nu_{35}\left(x,y,z,t\right)=a_{0}-\frac{12\sqrt{\alpha \mu }\cot\left(\sqrt{\alpha \mu }\left(\frac{t^{\alpha_{1}}\left(4\alpha \mu +s\right)}{\alpha_{1}r}+x+y+z+\vartheta \right)\right)}{p+q}. \end{array}$$

When [αμ<0]:

(56) $$\begin{array}{c} \nu_{36}\left(x,y,z,t\right)=a_{0}-\frac{12\sqrt{\alpha (-\mu )}\tanh\left(\sqrt{\alpha (-\mu )}\left(\frac{t^{\alpha_{1}}\left(4\alpha \mu +s\right)}{\alpha_{1}r}+x+y+z\right)\mp \frac{\log(\vartheta )}{2}\right)}{p+q}, \end{array}$$

(57) $$\begin{array}{c} \nu_{37}\left(x,y,z,t\right)=a_{0}-\frac{12\sqrt{\alpha (-\mu )}\coth\left(\sqrt{\alpha (-\mu )}\left(\frac{t^{\alpha_{1}}\left(4\alpha \mu +s\right)}{\alpha_{1}r}+x+y+z\right)\mp \frac{\log(\vartheta )}{2}\right)}{p+q}. \end{array}$$

When [α=0]:

Thus, when [λ>0], the solutions take the following forms:

(58) $$\begin{array}{c} \nu_{38}\left(x,y,z,t\right)=a_{0}+\frac{12\lambda (\frac{1}{\mu e^{\lambda (\frac{\left(s-\lambda^{2}\right)t^{\alpha_{1}}}{\alpha_{1}r}+x+y+z+\vartheta )}-1}+1)}{p+q}, \end{array}$$

(59) $$\begin{array}{c} \nu_{39}\left(x,y,z,t\right)=a_{0}+\frac{12\mu (1-\frac{1}{\mu e^{\lambda (\frac{\left(s-\lambda^{2}\right)t^{\alpha_{1}}}{\alpha_{1}r}+x+y+z+\vartheta )}+1})}{p+q}. \end{array}$$

When $\left[4\;\alpha \;\mu >\lambda^{2}\right]$: while [μ>0]:

(60) $$\begin{array}{c} \nu_{40}\left(x,y,z,t\right)=\frac{a_{0}\left(p+q\right)+6\lambda -6\sqrt{4\alpha \mu -\lambda^{2}}\tan\left(\frac{1}{2}\sqrt{4\alpha \mu -\lambda^{2}}\left(\frac{t^{\alpha_{1}}\left(4\alpha \mu -\lambda^{2}+s\right)}{\alpha_{1}r}+x+y+z+\vartheta \right)\right)}{p+q}, \end{array}$$

(61) $$\begin{array}{c} \nu_{41}\left(x,y,z,t\right)=\frac{a_{0}\left(p+q\right)+6\lambda -6\sqrt{4\alpha \mu -\lambda^{2}}\cot\left(\frac{1}{2}\sqrt{4\alpha \mu -\lambda^{2}}\left(\frac{t^{\alpha_{1}}\left(4\alpha \mu -\lambda^{2}+s\right)}{\alpha_{1}r}+x+y+z+\vartheta \right)\right)}{p+q}. \end{array}$$

When $\left[4\;\alpha \;\mu <\lambda^{2}\right]$:

Thus, when [μ>0], the solutions take the following forms:

(62) $$\begin{array}{c} \nu_{42}\left(x,y,z,t\right)=\frac{a_{0}\left(p+q\right)-6\left(\lambda +\sqrt{4\alpha \mu -\lambda^{2}}\tan\left(\frac{1}{2}\sqrt{4\alpha \mu -\lambda^{2}}\left(\frac{t^{\alpha_{1}}\left(4\alpha \mu -\lambda^{2}+s\right)}{\alpha_{1}r}+x+y+z+\vartheta \right)\right)\right)}{p+q}, \end{array}$$

(63) $$\begin{array}{c} \nu_{43}\left(x,y,z,t\right)=\frac{a_{0}\left(p+q\right)-6\left(\lambda +\sqrt{4\alpha \mu -\lambda^{2}}\cot\left(\frac{1}{2}\sqrt{4\alpha \mu -\lambda^{2}}\left(\frac{t^{\alpha_{1}}\left(4\alpha \mu -\lambda^{2}+s\right)}{\alpha_{1}r}+x+y+z+\vartheta \right)\right)\right)}{p+q}. \end{array}$$

*Family II*

$$a_{1}\to 0,a_{-1}\to \frac{12\alpha }{p+q},c\to -\frac{-4\alpha \mu +\lambda^{2}-s}{r},\mathrm{where}\;\left[r\neq 0,\;p+q\neq 0,\;\alpha \neq 0,\;-4\;\alpha \;\mu +\lambda^{2}\neq s\right].$$

According to the value of these parameters, the relevant traveling wave solutions of Equation (12) are given as follows:

When [λ=0]:

While [αμ>0]:

(64) $$\begin{array}{c} \nu_{44}\left(x,y,z,t\right)=a_{0}+\frac{12\sqrt{\alpha \mu }\cot\left(\sqrt{\alpha \mu }\left(\frac{t^{\alpha_{1}}\left(4\alpha \mu +s\right)}{\alpha_{1}r}+x+y+z+\vartheta \right)\right)}{p+q}, \end{array}$$

(65) $$\begin{array}{c} \nu_{45}\left(x,y,z,t\right)=a_{0}+\frac{12\sqrt{\alpha \mu }\tan\left(\sqrt{\alpha \mu }\left(\frac{t^{\alpha_{1}}\left(4\alpha \mu +s\right)}{\alpha_{1}r}+x+y+z+\vartheta \right)\right)}{p+q}. \end{array}$$

While [αμ<0]:

(66) $$\begin{array}{c} \nu_{46}\left(x,y,z,t\right)=a_{0}-\frac{12\sqrt{\alpha (-\mu )}\coth\left(\sqrt{\alpha (-\mu )}\left(\frac{t^{\alpha_{1}}\left(4\alpha \mu +s\right)}{\alpha_{1}r}+x+y+z\right)\mp \frac{\log(\vartheta )}{2}\right)}{p+q}, \end{array}$$

(67) $$\begin{array}{c} \nu_{47}\left(x,y,z,t\right)=a_{0}-\frac{12\sqrt{\alpha (-\mu )}\tanh\left(\sqrt{\alpha (-\mu )}\left(\frac{t^{\alpha_{1}}\left(4\alpha \mu +s\right)}{\alpha_{1}r}+x+y+z\right)\mp \frac{\log(\vartheta )}{2}\right)}{p+q}. \end{array}$$

When $\left[4\;\alpha \;\mu >\lambda^{2}\right]$:

Thus, when [μ>0], the solutions take the following forms:

(68) $$\begin{array}{c} \nu_{48}\left(x,y,z,t\right)=a_{0}-\frac{24\alpha \mu }{\left(p+q\right)(\lambda -\sqrt{4\alpha \mu -\lambda^{2}}\tan\left(\frac{1}{2}\sqrt{4\alpha \mu -\lambda^{2}}\left(\frac{t^{\alpha_{1}}\left(4\alpha \mu -\lambda^{2}+s\right)}{\alpha_{1}r}+x+y+z+\vartheta \right)\right))}, \end{array}$$

(69) $$\begin{array}{c} \nu_{49}\left(x,y,z,t\right)=a_{0}-\frac{24\alpha \mu }{\left(p+q\right)(\lambda -\sqrt{4\alpha \mu -\lambda^{2}}\cot\left(\frac{1}{2}\sqrt{4\alpha \mu -\lambda^{2}}\left(\frac{t^{\alpha_{1}}\left(4\alpha \mu -\lambda^{2}+s\right)}{\alpha_{1}r}+x+y+z+\vartheta \right)\right))}. \end{array}$$

When $\left[4\;\alpha \;\mu <\lambda^{2}\right]$:

While [μ>0]:

(70) $$\begin{array}{c} \nu_{50}\left(x,y,z,t\right)=a_{0}+\frac{24\alpha \mu }{\left(p+q\right)(\lambda +\sqrt{4\alpha \mu -\lambda^{2}}\tan\left(\frac{1}{2}\sqrt{4\alpha \mu -\lambda^{2}}\left(\frac{t^{\alpha_{1}}\left(4\alpha \mu -\lambda^{2}+s\right)}{\alpha_{1}r}+x+y+z+\vartheta \right)\right))}, \end{array}$$

(71) $$\begin{array}{c} \nu_{51}\left(x,y,z,t\right)=a_{0}+\frac{24\alpha \mu }{\left(p+q\right)(\lambda +\sqrt{4\alpha \mu -\lambda^{2}}\cot\left(\frac{1}{2}\sqrt{4\alpha \mu -\lambda^{2}}\left(\frac{t^{\alpha_{1}}\left(4\alpha \mu -\lambda^{2}+s\right)}{\alpha_{1}r}+x+y+z+\vartheta \right)\right))}. \end{array}$$

### 2.7. Utilization of the Generalized Riccati Expansion Method

Applying this method enables putting the general solution of Equation (13) in the next formula:

(72) $$\begin{array}{c} \nu \left(\Theta \right)=\sum_{i=1}^{N}a_{i}Q{\left(\Theta \right)}^{i}+\sum_{i=1}^{N}b_{i}Q{\left(\Theta \right)}^{-i}+a_{0}=a_{1}Q\left(\Theta \right)+a_{0}+\frac{b_{1}}{Q(\Theta )}, \end{array}$$

where $a_{0},\;a_{i},\;b_{i},\;\left\{i=0,1,\cdots ,N\right\}$ and Q(Θ) is the solution of the next ODE

$$\begin{array}{c} Q^{\prime }\left(\Theta \right)=A+BQ\left(\Theta \right)+mQ{\left(\Theta \right)}^{2}, \end{array}$$

where A,B,andm are arbitrary constants. Handling of Equation (13) by utilizing Equation (72) converts the left-hand side of Equation (13) to polynomial function of Q(Θ). Gather all coefficients of terms that have the same degree and equate them to zero. Solving the obtained system of equation leads to

$$b_{1}\to \frac{12A}{p+q},a_{1}\to 0,c\to \frac{4Am-B^{2}+s}{r},\;\mathrm{where}\;\left[p+q\neq 0,\;A\neq 0,\;r\neq 0,\;4\;A\;m-B^{2}+s\neq 0\right].$$

According to the value of these parameters, we get the relevant traveling wave solutions of Equation (12):

When $\left[\Delta =\left(B^{2}-4\;m\;A\right)>0\;\&\;m\;B\neq 0\;\&\;m\;A\neq 0\;\&\;F^{2}-R^{2}>0\right]$:

(73) $$\begin{array}{c} \nu_{52}\left(x,y,z,t\right)=a_{0}-\frac{24Am}{\left(p+q\right)(\sqrt{\Delta }\tanh\left(\frac{1}{2}\sqrt{\Delta }\left(\frac{t^{\alpha_{1}}\left(4Am-B^{2}+s\right)}{\alpha_{1}r}+x+y+z\right)\right)+B)}, \end{array}$$

(74) $$\begin{array}{c} \nu_{53}\left(x,y,z,t\right)=a_{0}-\frac{24Am}{\left(p+q\right)(\sqrt{\Delta }\coth\left(\frac{1}{2}\sqrt{\Delta }\left(\frac{t^{\alpha_{1}}\left(4Am-B^{2}+s\right)}{\alpha_{1}r}+x+y+z\right)\right)+B)}, \end{array}$$

(75) $$\begin{array}{c} \nu_{54}\left(x,y,z,t\right)=a_{0}-\frac{24Am}{\left(p+q\right)(B+\sqrt{\Delta }\left(\tanh\left(\sqrt{\Delta }\left(\frac{t^{\alpha_{1}}\left(4Am-B^{2}+s\right)}{\alpha_{1}r}+x+y+z\right)\right)\pm i\sec h\left(\sqrt{\Delta }\left(\frac{t^{\alpha_{1}}\left(4Am-B^{2}+s\right)}{\alpha_{1}r}+x+y+z\right)\right)\right))}, \end{array}$$

(76) $$\begin{array}{c} \nu_{55}\left(x,y,z,t\right)=a_{0}-\frac{24Am}{\left(p+q\right)(\sqrt{\Delta }\left(\coth\left(\sqrt{\Delta }\left(\frac{t^{\alpha_{1}}\left(4Am-B^{2}+s\right)}{\alpha_{1}r}+x+y+z\right)\right)\pm \csc h\left(\sqrt{\Delta }\left(\frac{t^{\alpha_{1}}\left(4Am-B^{2}+s\right)}{\alpha_{1}r}+x+y+z\right)\right)\right)+B)}, \end{array}$$

(77) $$\begin{array}{c} \nu_{56}\left(x,y,z,t\right)=a_{0}-\frac{48Am}{\left(p+q\right)(\sqrt{\Delta }\left(\tanh\left(\frac{1}{4}\sqrt{\Delta }\left(\frac{t^{\alpha_{1}}\left(4Am-B^{2}+s\right)}{\alpha_{1}r}+x+y+z\right)\right)+\coth\left(\frac{1}{4}\sqrt{\Delta }\left(\frac{t^{\alpha_{1}}\left(4Am-B^{2}+s\right)}{\alpha_{1}r}+x+y+z\right)\right)\right)+2B)}, \end{array}$$

(78) $$\begin{array}{c} \nu_{57}\left(x,y,z,t\right)=a_{0}-\frac{24Am}{\left(p+q\right)\left(\frac{\sqrt{\Delta }R\cosh\left(\sqrt{\Delta }\left(\frac{t^{\alpha_{1}}\left(4Am-B^{2}+s\right)}{\alpha_{1}r}+x+y+z\right)\right)-\sqrt{\Delta (F^{2}+R^{2})}}{R\sinh(\sqrt{\Delta }\left(\frac{t^{\alpha_{1}}\left(4Am-B^{2}+s\right)}{\alpha_{1}r}+x+y+z\right))+F}+B\right)}, \end{array}$$

(79) $$\begin{array}{c} \nu_{58}\left(x,y,z,t\right)=a_{0}-\frac{24Am}{\left(p+q\right)\left(\frac{\sqrt{\Delta }R\sinh\left(\sqrt{\Delta }\left(\frac{t^{\alpha_{1}}\left(4Am-B^{2}+s\right)}{\alpha_{1}r}+x+y+z\right)\right)+\sqrt{\Delta (R^{2}-F^{2})}}{R\cosh(\sqrt{\Delta }\left(\frac{t^{\alpha_{1}}\left(4Am-B^{2}+s\right)}{\alpha_{1}r}+x+y+z\right))+F}+B\right)}, \end{array}$$

(80) $$\begin{array}{c} \nu_{59}\left(x,y,z,t\right)=\frac{a_{0}\left(p+q\right)+6\sqrt{\Delta }\tanh\left(\frac{1}{2}\sqrt{\Delta }\left(\frac{t^{\alpha_{1}}\left(4Am-B^{2}+s\right)}{\alpha_{1}r}+x+y+z\right)\right)-6F}{p+q}, \end{array}$$

(81) $$\begin{array}{c} \nu_{60}\left(x,y,z,t\right)=a_{0}-\frac{6(B-\sqrt{\Delta }\coth\left(\frac{1}{2}\sqrt{\Delta }\left(\frac{t^{\alpha_{1}}\left(4Am-B^{2}+s\right)}{\alpha_{1}r}+x+y+z\right)\right))}{p+q}, \end{array}$$

(82) $$\begin{array}{c} \nu_{61}\left(x,y,z,t\right)=\frac{6\sec h(\sqrt{\Delta }\left(\frac{t^{\alpha_{1}}\left(4Am-B^{2}+s\right)}{\alpha_{1}r}+x+y+z\right))}{p+q}\\ \times \left[\sqrt{\Delta }\sinh\left(\sqrt{\Delta }\left(\frac{t^{\alpha_{1}}\left(4Am-B^{2}+s\right)}{\alpha_{1}r}+x+y+z\right)\right)-\left(B\cosh\left(\sqrt{\Delta }\left(\frac{t^{\alpha_{1}}\left(4Am-B^{2}+s\right)}{\alpha_{1}r}+x+y+z\right)\right)\pm i\sqrt{\Delta }\right)\right]+a_{0}, \end{array}$$

(83) $$\begin{array}{c} \nu_{62}\left(x,y,z,t\right)=a_{0}-\frac{6(B-\csc h\left(\sqrt{\Delta }\left(\frac{t^{\alpha_{1}}\left(4Am-B^{2}+s\right)}{\alpha_{1}r}+x+y+z\right)\right)\left(\sqrt{\Delta }\cosh\left(\sqrt{\Delta }\left(\frac{t^{\alpha_{1}}\left(4Am-B^{2}+s\right)}{\alpha_{1}r}+x+y+z\right)\right)\pm \sqrt{\Delta }\right))}{p+q}, \end{array}$$

(84) $$\begin{array}{c} \nu_{63}\left(x,y,z,t\right)=\frac{a_{0}\left(p+q\right)+3\sqrt{\Delta }\tanh\left(\frac{1}{2}\sqrt{\Delta }\left(\frac{t^{\alpha_{1}}\left(4Am-B^{2}+s\right)}{\alpha_{1}r}+x+y+z\right)\right)\left(\coth^{2}\left(\frac{1}{2}\sqrt{\Delta }\left(\frac{t^{\alpha_{1}}\left(4Am-B^{2}+s\right)}{\alpha_{1}r}+x+y+z\right)\right)+1\right)-6B}{p+q}. \end{array}$$

When $\left[\Delta =\left(B^{2}-4\;m\;A\right)<0\;\&\;m\;B\neq 0\;\&\;m\;A\neq 0\;\&\;F^{2}-R^{2}<0\right]$:

(85) $$\begin{array}{c} \nu_{64}\left(x,y,z,t\right)=a_{0}-\frac{24Am}{\left(p+q\right)(\sqrt{\Delta }\tanh\left(\frac{1}{2}\sqrt{\Delta }\left(\frac{t^{\alpha_{1}}\left(4Am-B^{2}+s\right)}{\alpha_{1}r}+x+y+z\right)\right)+B)}, \end{array}$$

(86) $$\begin{array}{c} \nu_{65}\left(x,y,z,t\right)=a_{0}-\frac{24Am}{\left(p+q\right)(\sqrt{\Delta }\coth\left(\frac{1}{2}\sqrt{\Delta }\left(\frac{t^{\alpha_{1}}\left(4Am-B^{2}+s\right)}{\alpha_{1}r}+x+y+z\right)\right)+B)}, \end{array}$$

(87) $$\begin{array}{c} \nu_{66}\left(x,y,z,t\right)=a_{0}-\frac{24Am}{\left(p+q\right)(B-\sqrt{-\Delta }\left(\tan\left(\sqrt{-\Delta }\left(\frac{t^{\alpha_{1}}\left(4Am-B^{2}+s\right)}{\alpha_{1}r}+x+y+z\right)\right)\pm \sec h\left(\sqrt{\Delta }\left(\frac{t^{\alpha_{1}}\left(4Am-B^{2}+s\right)}{\alpha_{1}r}+x+y+z\right)\right)\right))}, \end{array}$$

(88) $$\begin{array}{c} \nu_{67}\left(x,y,z,t\right)=a_{0}-\frac{24Am}{\left(p+q\right)(\sqrt{-\Delta }\left(\cot\left(\sqrt{-\Delta }\left(\frac{t^{\alpha_{1}}\left(4Am-B^{2}+s\right)}{\alpha_{1}r}+x+y+z\right)\right)\pm \csc\left(\sqrt{-\Delta }\left(\frac{t^{\alpha_{1}}\left(4Am-B^{2}+s\right)}{\alpha_{1}r}+x+y+z\right)\right)\right)+B)}, \end{array}$$

(89) $$\begin{array}{c} \nu_{68}\left(x,y,z,t\right)=a_{0}-\frac{48Am}{\left(p+q\right)(\sqrt{\Delta }\tanh\left(\frac{1}{4}\sqrt{\Delta }\left(\frac{t^{\alpha_{1}}\left(4Am-B^{2}+s\right)}{\alpha_{1}r}+x+y+z\right)\right)\left(\coth^{2}\left(\frac{1}{4}\sqrt{\Delta }\left(\frac{t^{\alpha_{1}}\left(4Am-B^{2}+s\right)}{\alpha_{1}r}+x+y+z\right)\right)+1\right)+2B)}, \end{array}$$

(90) $$\begin{array}{c} \nu_{69}\left(x,y,z,t\right)=a_{0}-\frac{24Am}{\left(p+q\right)\left(\frac{\sqrt{-\Delta }R\cosh\left(\sqrt{\Delta }\left(\frac{t^{\alpha_{1}}\left(4Am-B^{2}+s\right)}{\alpha_{1}r}+x+y+z\right)\right)-\sqrt{\Delta (F-R)(F+R)}}{R\sin(\sqrt{-\Delta }\left(\frac{t^{\alpha_{1}}\left(4Am-B^{2}+s\right)}{\alpha_{1}r}+x+y+z\right))+F}+B\right)}, \end{array}$$

(91) $$\begin{array}{c} \nu_{70}\left(x,y,z,t\right)=a_{0}-\frac{24Am}{\left(p+q\right)\left(\frac{\sqrt{\Delta (F-R)(F+R)}-\sqrt{\Delta }R\sinh\left(\sqrt{\Delta }\left(\frac{t^{\alpha_{1}}\left(4Am-B^{2}+s\right)}{\alpha_{1}r}+x+y+z\right)\right)}{R\cosh(\sqrt{\Delta }\left(\frac{t^{\alpha_{1}}\left(4Am-B^{2}+s\right)}{\alpha_{1}r}+x+y+z\right))+F}+B\right)}, \end{array}$$

(92) $$\begin{array}{c} \nu_{71}\left(x,y,z,t\right)=\frac{a_{0}\left(p+q\right)+6\sqrt{\Delta }\tanh\left(\frac{1}{2}\sqrt{\Delta }\left(\frac{t^{\alpha_{1}}\left(4Am-B^{2}+s\right)}{\alpha_{1}r}+x+y+z\right)\right)-6B}{p+q}, \end{array}$$

(93) $$\begin{array}{c} \nu_{72}\left(x,y,z,t\right)=a_{0}-\frac{6(B-\sqrt{\Delta }\coth\left(\frac{1}{2}\sqrt{\Delta }\left(\frac{t^{\alpha_{1}}\left(4Am-B^{2}+s\right)}{\alpha_{1}r}+x+y+z\right)\right))}{p+q}, \end{array}$$

(94) $$\begin{array}{c} \nu_{73}\left(x,y,z,t\right)=\frac{6\sec h(\sqrt{\Delta }\left(\frac{t^{\alpha_{1}}\left(4Am-B^{2}+s\right)}{\alpha_{1}r}+x+y+z\right))}{p+q}\\ \times \left[\sqrt{\Delta }\sinh\left(\sqrt{\Delta }\left(\frac{t^{\alpha_{1}}\left(4Am-B^{2}+s\right)}{\alpha_{1}r}+x+y+z\right)\right)-\left(B\cosh\left(\sqrt{\Delta }\left(\frac{t^{\alpha_{1}}\left(4Am-B^{2}+s\right)}{\alpha_{1}r}+x+y+z\right)\right)\pm \sqrt{-\Delta }\right)\right]+a_{0}, \end{array}$$

(95) $$\begin{array}{c} \nu_{74}\left(x,y,z,t\right)=a_{0}-\frac{6(B-\csc\left(\sqrt{-\Delta }\left(\frac{t^{\alpha_{1}}\left(4Am-B^{2}+s\right)}{\alpha_{1}r}+x+y+z\right)\right)\left(\sqrt{-\Delta }\cos\left(\sqrt{\Delta }\left(\frac{t^{\alpha_{1}}\left(4Am-B^{2}+s\right)}{\alpha_{1}r}+x+y+z\right)\right)\pm \sqrt{\Delta }\right))}{p+q}, \end{array}$$

(96) $$\begin{array}{c} \nu_{75}\left(x,y,z,t\right)=\frac{a_{0}\left(p+q\right)+3\sqrt{\Delta }\tanh\left(\frac{1}{2}\sqrt{\Delta }\left(\frac{t^{\alpha_{1}}\left(4Am-B^{2}+s\right)}{\alpha_{1}r}+x+y+z\right)\right)\left(\coth^{2}\left(\frac{1}{2}\sqrt{\Delta }\left(\frac{t^{\alpha_{1}}\left(4Am-B^{2}+s\right)}{\alpha_{1}r}+x+y+z\right)\right)+1\right)-6B}{p+q}. \end{array}$$

### 2.8. Utilization of the Generalized Sinh–Gordon Expansion Method

Applying this method enables putting the general solution of Equation (13) in the next formula:

(97) $$\begin{array}{c} \nu \left(\Theta \right)=\sum_{i=1}^{N}cosh^{i-1}\left(w\left(\Theta \right)\right)[B_{i}\;sinh\left(w\left(\Theta \right)\right)+A_{i}\;cosh\left(w\left(\Theta \right)\right)]+A_{0}=A_{1}\cosh\left(w\left(\Theta \right)\right)+A_{0}+B_{1}\sinh\left(w\left(\Theta \right)\right), \end{array}$$

where $A_{1},A_{0},\mathrm{and}\;B_{1}$ are arbitrary constants. Handling of Equation (13) by utilizing Equation (97) and its derivatives converts the left-hand side of Equation (13) to polynomial function of f(Θ). Gather all coefficients of terms that have the same degree and equate them to zero. Solving the obtained system of equation leads to

**Case I**: When $\left[w^{\prime }\left(\Theta \right)=\sinh\left(w\left(\Theta \right)\right)\right]$:

*Family I*:

$$A_{1}\to -\frac{6}{p+q},B_{1}\to -\frac{6}{\sqrt{p^{2}+2pq+q^{2}}},c\to \frac{s-1}{r},\;\mathrm{where}\;\left[p+q\neq 0,\;r\neq 0,\;p^{2}+2pq+q^{2}\neq 0\right].$$

According to the value of these parameters, the relevant traveling wave solutions of Equation (12) are given in the following formulas:

(98) $$\begin{array}{c} \nu_{76}\left(x,y,z,t\right)=A_{0}+\frac{6\tanh(\frac{\left(s-1\right)t^{\alpha_{1}}}{\alpha_{1}r}+x+y+z)}{p+q}-\frac{6\left(\pm i\right)\sec h\left(\frac{\left(s-1\right)t^{\alpha_{1}}}{\alpha_{1}r}+x+y+z\right)}{\sqrt{{\left(p+q\right)}^{2}}}, \end{array}$$

(99) $$\begin{array}{c} \nu_{77}\left(x,y,z,t\right)=A_{0}+\frac{6\coth(\frac{\left(s-1\right)t^{\alpha_{1}}}{\alpha_{1}r}+x+y+z)}{p+q}-\frac{6\left(\pm i\right)\csc h\left(\frac{\left(s-1\right)t^{\alpha_{1}}}{\alpha_{1}r}+x+y+z\right)}{\sqrt{{\left(p+q\right)}^{2}}}. \end{array}$$

*Family II*:

$$A_{1}\to 0,B_{1}\to -\frac{12}{p+q},c\to {\left(\frac{s}{r}\right)}^{2},\;\mathrm{where},\;\left[p+q\neq 0,\;r\neq 0\right].$$

According to the value of these parameters, the relevant traveling wave solutions of Equation (12) are given in the following formulas:

(100) $$\begin{array}{c} \nu_{78}\left(x,y,z,t\right)=A_{0}+\frac{12\tanh(\frac{\left(s-4\right)t^{\alpha_{1}}}{\alpha_{1}r}+x+y+z)}{p+q}, \end{array}$$

(101) $$\begin{array}{c} \nu_{79}\left(x,y,z,t\right)=A_{0}+\frac{12\coth(\frac{\left(s-4\right)t^{\alpha_{1}}}{\alpha_{1}r}+x+y+z)}{p+q}. \end{array}$$

**Case II**: When $\left[w^{\prime }\left(\xi \right)=\cosh\left(w\left(\xi \right)\right)\right]$:

*Family I*:

$$A_{1}\to 0,B_{1}\to -\frac{12}{p+q},c\to \frac{s}{r},\;\mathrm{where},\;\left[p+q\neq 0,\;r\neq 0\right].$$

According to the value of these parameters, the relevant traveling wave solutions of Equation (12) are given by the following formulas:

(102) $$\begin{array}{c} \nu_{80}\left(x,y,z,t\right)=A_{0}-\frac{12\tan(\frac{st^{\alpha_{1}}}{\alpha_{1}r}+x+y+z)}{p+q}, \end{array}$$

(103) $$\begin{array}{c} \nu_{81}\left(x,y,z,t\right)=A_{0}+\frac{12\cot(\frac{s^{2}t^{\alpha_{1}}}{\alpha_{1}r^{2}}+x+y+z)}{p+q}. \end{array}$$

### 2.9. Utilization of Riccati–Bernoulli Sub-ODE Method

Applying this method enables putting the general solution of Equation (13) in the next formula:

(104) $$\begin{array}{c} \nu \left(\xi \right)=a\nu {\left(\Theta \right)}^{2-m}+b\nu \left(\Theta \right)+\eta \nu {\left(\Theta \right)}^{m}, \end{array}$$

where a,b,η,andm are arbitrary constants. Substituting Equation (104) and its derivatives into Equation (13), in addition to collecting all coefficients of the same term of ν(ξ), we get the system of equation. Solving this system leads to

*Family I*:

$$a\to \frac{1}{12}\left(-p-q\right),b\to -\sqrt{s-cr},\eta \to 0,\;\mathrm{where}\;\left[s-cr>0\right].$$

Thus, the solitary wave solutions of Equation (12) are given by:

(105) $$\begin{array}{c} \nu_{82}\left(x,y,z,t\right)={\left(\mu e^{\sqrt{s-cr}\left(\frac{ct^{\alpha_{1}}}{\alpha_{1}}+x+y+z\right)}-\frac{p+q}{12\sqrt{s-cr}}\right)}^{-r}. \end{array}$$

*Family II*:

$$a\to \frac{1}{12}\left(-p-q\right),b\to \sqrt{s-cr},\eta \to 0,\;\mathrm{where}\;\left[s-cr>0\right].$$

Thus, the solitary wave solutions of Equation (12) are given by

(106) $$\begin{array}{c} \nu_{83}\left(x,y,z,t\right)={\left(\frac{p+q}{12\sqrt{s-cr}}+\mu e^{-\sqrt{s-cr}\left(\frac{ct^{\alpha_{1}}}{\alpha_{1}}+x+y+z\right)}\right)}^{-r}. \end{array}$$

*Family III*:

$$a\to \frac{1}{12}\left(-p-q\right),b\to 0,c\to \frac{3(s-\eta r)}{p+q},\;\mathrm{where}\;\left[s-cr>0\right].$$

Thus, the solitary wave solutions of Equation (12) are given by

When $\left[m\neq 0,\;a\neq 0,\;b^{2}-4\;a\;\eta <0\right]$

(107) $$\begin{array}{c} \nu_{84}\left(x,y,z,t\right)=\frac{\left(p+q\right)\tan(\frac{1}{2}\sqrt{cr-s}\left(\frac{ct^{\alpha_{1}}}{\alpha_{1}}+x+y+z+\vartheta \right))}{6\sqrt{cr-s}}, \end{array}$$

(108) $$\begin{array}{c} \nu_{85}\left(x,y,z,t\right)=-\frac{\left(p+q\right)\cot(\frac{1}{2}\sqrt{cr-s}\left(\frac{ct^{\alpha_{1}}}{\alpha_{1}}+x+y+z+\vartheta \right))}{6\sqrt{cr-s}}. \end{array}$$

When $\left[m\neq 0,\;a\neq 0,\;b^{2}-4\;a\;\eta >0\right]$

(109) $$\begin{array}{c} \nu_{86}\left(x,y,z,t\right)=\frac{\left(p+q\right)\coth(\frac{1}{2}\sqrt{s-cr}\left(\frac{ct^{\alpha_{1}}}{\alpha_{1}}+x+y+z+\vartheta \right))}{6\sqrt{cr-s}}, \end{array}$$

(110) $$\begin{array}{c} \nu_{87}\left(x,y,z,t\right)=\frac{\left(p+q\right)\tanh(\frac{1}{2}\sqrt{s-cr}\left(\frac{ct^{\alpha_{1}}}{\alpha_{1}}+x+y+z+\vartheta \right))}{6\sqrt{cr-s}}. \end{array}$$

### 2.10. Utilization of the Modified Auxiliary Method

Applying this method enables putting the general solution of Equation (13) in the next formula:

(111) $$\begin{array}{c} \nu \left(\Theta \right)=\sum_{i=1}^{N}a_{i}\;K^{if(\Theta )}+\sum_{i=1}^{N}b_{i}\;K^{-if(\Theta )}+a_{0}=a_{1}\;K^{f(\Theta )}+a_{0}+b_{1}\;K^{-f(\Theta )}, \end{array}$$

where $a_{i},\;b_{i}$ are arbitrary constants and f(Θ) is the solution of the next ODE

$$\begin{array}{c} f^{\prime }\left(\Theta \right)=\frac{\beta +\alpha K^{-f(\Theta )}+\sigma K^{f(\Theta )}}{\ln(K)}, \end{array}$$

where β,α,andσ are arbitrary constants. Handling of Equation (13) by utilizing Equation (111) and its derivatives converts the left-hand side of Equation (13) to polynomial function of $K^{f(\Theta )}$. Gather all coefficients of terms that have the same degree and equate them to zero. Solving the obtained system of equation yields:

**Family I:**

$$a_{1}\to 0,b_{1}\to \frac{12\alpha }{p+q},c\to \frac{4\alpha \sigma -\beta^{2}+s}{r},\;\mathrm{where}\;\left[p+q\neq 0,\;r\neq 0,\;4\alpha \sigma -\beta^{2}+s\neq 0,\;\alpha \neq 0\right].$$

According to the value of these parameters, the relevant traveling wave solutions of Equation (12) are given as follows:

When $\left[\beta^{2}-4\;\alpha \;\sigma <0\;\&\;\sigma \neq 0\right]$:

(112) $$\begin{array}{c} \nu_{88}\left(x,y,z,t\right)=a_{0}-\frac{24\alpha \sigma }{\left(p+q\right)(\beta -\sqrt{4\alpha \sigma -\beta^{2}}\tan\left(\frac{1}{2}\sqrt{4\alpha \sigma -\beta^{2}}\left(\frac{t^{\alpha_{1}}\left(4\alpha \sigma -\beta^{2}+s\right)}{r\alpha_{1}}+x+y+z\right)\right))}, \end{array}$$

(113) $$\begin{array}{c} \nu_{89}\left(x,y,z,t\right)=a_{0}-\frac{24\alpha \sigma }{\left(p+q\right)(\beta -\sqrt{4\alpha \sigma -\beta^{2}}\cot\left(\frac{1}{2}\sqrt{4\alpha \sigma -\beta^{2}}\left(\frac{t^{\alpha_{1}}\left(4\alpha \sigma -\beta^{2}+s\right)}{r\alpha_{1}}+x+y+z\right)\right))}. \end{array}$$

When $\left[\beta^{2}-4\;\alpha \;\sigma >0\;\&\;\sigma \neq 0\right]$:

(114) $$\begin{array}{c} \nu_{90}\left(x,y,z,t\right)=a_{0}-\frac{24\alpha \sigma }{\left(p+q\right)(\beta +\sqrt{\beta^{2}-4\alpha \sigma }\tanh\left(\frac{1}{2}\sqrt{\beta^{2}-4\alpha \sigma }\left(\frac{t^{\alpha_{1}}\left(4\alpha \sigma -\beta^{2}+s\right)}{r\alpha_{1}}+x+y+z\right)\right))}, \end{array}$$

(115) $$\begin{array}{c} \nu_{91}\left(x,y,z,t\right)=a_{0}-\frac{24\alpha \sigma }{\left(p+q\right)(\beta +\sqrt{\beta^{2}-4\alpha \sigma }\coth\left(\frac{1}{2}\sqrt{\beta^{2}-4\alpha \sigma }\left(\frac{t^{\alpha_{1}}\left(4\alpha \sigma -\beta^{2}+s\right)}{r\alpha_{1}}+x+y+z\right)\right))}. \end{array}$$

When $\left[\beta^{2}+4\;\alpha^{2}<0\;\&\;\alpha =-\sigma \;\&\;\sigma \neq 0\right]$:

(116) $$\begin{array}{c} \nu_{92}\left(x,y,z,t\right)=a_{0}+\frac{24\alpha^{2}}{\left(p+q\right)(\beta -\sqrt{-4\alpha^{2}-\beta^{2}}\tan\left(\frac{1}{2}\sqrt{-4\alpha^{2}-\beta^{2}}\left(\frac{t^{\alpha_{1}}\left(-4\alpha^{2}-\beta^{2}+s\right)}{r\alpha_{1}}+x+y+z\right)\right))}, \end{array}$$

(117) $$\begin{array}{c} \nu_{93}\left(x,y,z,t\right)=a_{0}+\frac{24\alpha^{2}}{\left(p+q\right)(\beta -\sqrt{-4\alpha^{2}-\beta^{2}}\cot\left(\frac{1}{2}\sqrt{-4\alpha^{2}-\beta^{2}}\left(\frac{t^{\alpha_{1}}\left(-4\alpha^{2}-\beta^{2}+s\right)}{r\alpha_{1}}+x+y+z\right)\right))}. \end{array}$$

When $\left[\beta^{2}+4\;\alpha^{2}>0\;\&\;\alpha =-\sigma \;\&\;\sigma \neq 0\right]$:

(118) $$\begin{array}{c} \nu_{94}\left(x,y,z,t\right)=a_{0}+\frac{24\alpha^{2}}{\left(p+q\right)(\beta +\sqrt{4\alpha^{2}+\beta^{2}}\tanh\left(\frac{1}{2}\sqrt{4\alpha^{2}+\beta^{2}}\left(\frac{t^{\alpha_{1}}\left(-4\alpha^{2}-\beta^{2}+s\right)}{r\alpha_{1}}+x+y+z\right)\right))}, \end{array}$$

(119) $$\begin{array}{c} \nu_{95}\left(x,y,z,t\right)=a_{0}+\frac{24\alpha^{2}}{\left(p+q\right)(\beta +\sqrt{4\alpha^{2}+\beta^{2}}\coth\left(\frac{1}{2}\sqrt{4\alpha^{2}+\beta^{2}}\left(\frac{t^{\alpha_{1}}\left(-4\alpha^{2}-\beta^{2}+s\right)}{r\alpha_{1}}+x+y+z\right)\right))}. \end{array}$$

When $\left[\beta^{2}-4\;\alpha^{2}<0\;\&\;\alpha =\sigma \;\&\;\sigma \neq 0\right]$:

(120) $$\begin{array}{c} \nu_{96}\left(x,y,z,t\right)=a_{0}-\frac{24\alpha^{2}}{\left(p+q\right)(\beta -\sqrt{4\alpha^{2}-\beta^{2}}\tan\left(\frac{1}{2}\sqrt{4\alpha^{2}-\beta^{2}}\left(\frac{t^{\alpha_{1}}\left(4\alpha^{2}-\beta^{2}+s\right)}{r\alpha_{1}}+x+y+z\right)\right))}, \end{array}$$

(121) $$\begin{array}{c} \nu_{97}\left(x,y,z,t\right)=a_{0}-\frac{24\alpha^{2}}{\left(p+q\right)(\beta -\sqrt{4\alpha^{2}-\beta^{2}}\cot\left(\frac{1}{2}\sqrt{4\alpha^{2}-\beta^{2}}\left(\frac{t^{\alpha_{1}}\left(4\alpha^{2}-\beta^{2}+s\right)}{r\alpha_{1}}+x+y+z\right)\right))}. \end{array}$$

When $\left[\beta^{2}-4\;\alpha^{2}>0\;\&\;\alpha =\sigma \;\&\;\sigma \neq 0\right]$:

(122) $$\begin{array}{c} \nu_{98}\left(x,y,z,t\right)=a_{0}-\frac{24\alpha^{2}}{\left(p+q\right)(\beta +\sqrt{\beta^{2}-4\alpha^{2}}\tanh\left(\frac{1}{2}\sqrt{\beta^{2}-4\alpha^{2}}\left(\frac{t^{\alpha_{1}}\left(4\alpha^{2}-\beta^{2}+s\right)}{r\alpha_{1}}+x+y+z\right)\right))}, \end{array}$$

(123) $$\begin{array}{c} \nu_{99}\left(x,y,z,t\right)=a_{0}-\frac{24\alpha^{2}}{\left(p+q\right)(\beta +\sqrt{\beta^{2}-4\alpha^{2}}\coth\left(\frac{1}{2}\sqrt{\beta^{2}-4\alpha^{2}}\left(\frac{t^{\alpha_{1}}\left(4\alpha^{2}-\beta^{2}+s\right)}{r\alpha_{1}}+x+y+z\right)\right))}. \end{array}$$

When [ασ<0&α≠0&β=0]:

(124) $$\begin{array}{c} \nu_{100}\left(x,y,z,t\right)=a_{0}+\frac{12\sqrt{\alpha \sigma }\cot\left(\sqrt{\alpha \sigma }\left(\frac{t^{\alpha_{1}}\left(4\alpha \sigma +s\right)}{r\alpha_{1}}+x+y+z\right)\right)}{p+q}, \end{array}$$

(125) $$\begin{array}{c} \nu_{101}\left(x,y,z,t\right)=a_{0}-\frac{12\sqrt{\alpha \sigma }\tan\left(\sqrt{\alpha \sigma }\left(\frac{t^{\alpha_{1}}\left(4\alpha \sigma +s\right)}{r\alpha_{1}}+x+y+z\right)\right)}{p+q}. \end{array}$$

When [ασ>0&α≠0&β=0]:

(126) $$\begin{array}{c} \nu_{102}\left(x,y,z,t\right)=a_{0}+\frac{12\sqrt{-\alpha \sigma }\coth\left(\sqrt{-\alpha \sigma }\left(\frac{t^{\alpha_{1}}\left(4\alpha \sigma +s\right)}{r\alpha_{1}}+x+y+z\right)\right)}{p+q}, \end{array}$$

(127) $$\begin{array}{c} \nu_{103}\left(x,y,z,t\right)=a_{0}+\frac{12\sqrt{-\alpha \sigma }\tanh\left(\sqrt{-\alpha \sigma }\left(\frac{t^{\alpha_{1}}\left(4\alpha \sigma +s\right)}{r\alpha_{1}}+x+y+z\right)\right)}{p+q}. \end{array}$$

When [β=0&α=−σ]:

(128) $$\begin{array}{c} \nu_{104}\left(x,y,z,t\right)=a_{0}+\frac{12\alpha \tanh(\alpha \left(\frac{\left(s-4\alpha^{2}\right)t^{\alpha_{1}}}{r\alpha_{1}}+x+y+z\right))}{p+q}. \end{array}$$

When [β=κ&α=2κ&σ=0]:

(129) $$\begin{array}{c} \nu_{105}\left(x,y,z,t\right)=a_{0}+\frac{24\kappa }{\left(p+q\right)\left(e^{\kappa (\frac{\left(s-\kappa^{2}\right)t^{\alpha_{1}}}{r\alpha_{1}}+x+y+z)}-2\right)}. \end{array}$$

When [β=σ=0]:

(130) $$\begin{array}{c} \nu_{106}\left(x,y,z,t\right)=a_{0}+\frac{12r\alpha_{1}}{\left(p+q\right)(r\alpha_{1}\left(x+y+z\right)+st^{\alpha_{1}})}. \end{array}$$

When [β=0&α=σ]:

(131) $$\begin{array}{c} \nu_{107}\left(x,y,z,t\right)=a_{0}+\frac{12\alpha \cot(C+\alpha \left(\frac{\left(4\alpha^{2}+s\right)t^{\alpha_{1}}}{r\alpha_{1}}+x+y+z\right))}{p+q}. \end{array}$$

When [σ=0]:

(132) $$\begin{array}{c} \nu_{108}\left(x,y,z,t\right)=a_{0}-\frac{12\alpha \beta }{\left(p+q\right)\left(\alpha -\beta e^{\beta (\frac{\left(s-\beta^{2}\right)t^{\alpha_{1}}}{r\alpha_{1}}+x+y+z)}\right)}. \end{array}$$

When $\left[\beta^{2}-4\;\alpha \;\sigma =0\right]$:

(133) $$\begin{array}{c} \nu_{109}\left(x,y,z,t\right)=a_{0}+\frac{24\alpha \sigma (r\alpha_{1}\left(x+y+z\right)+st^{\alpha_{1}})}{\left(p+q\right)(-r\alpha_{1}\left(2\sqrt{\alpha \sigma }\left(x+y+z\right)+2\right)-2s\sqrt{\alpha \sigma }t^{\alpha_{1}})}. \end{array}$$

**Family II:**

$$a_{1}\to -\frac{12\sigma }{p+q},b_{1}\to 0,c\to \frac{4\alpha \sigma -\beta^{2}+s}{r},\;\mathrm{where}\;\left[\sigma \neq 0,\;p+q\neq 0,\;4\alpha \sigma -\beta^{2}+s\neq 0,\;r\neq 0\right].$$

According to the value of these parameters, the relevant traveling wave solutions of Equation (12) are given as follows:

When $\left[\beta^{2}-4\;\alpha \;\sigma <0\;\&\;\sigma \neq 0\right]$:

(134) $$\begin{array}{c} \nu_{110}\left(x,y,z,t\right)=\frac{a_{0}\left(p+q\right)+6\beta -6\sqrt{4\alpha \sigma -\beta^{2}}\tan\left(\frac{1}{2}\sqrt{4\alpha \sigma -\beta^{2}}\left(\frac{t^{\alpha_{1}}\left(4\alpha \sigma -\beta^{2}+s\right)}{r\alpha_{1}}+x+y+z\right)\right)}{p+q}, \end{array}$$

(135) $$\begin{array}{c} \nu_{111}\left(x,y,z,t\right)=\frac{a_{0}\left(p+q\right)+6\beta -6\sqrt{4\alpha \sigma -\beta^{2}}\cot\left(\frac{1}{2}\sqrt{4\alpha \sigma -\beta^{2}}\left(\frac{t^{\alpha_{1}}\left(4\alpha \sigma -\beta^{2}+s\right)}{r\alpha_{1}}+x+y+z\right)\right)}{p+q}. \end{array}$$

When $\left[\beta^{2}-4\;\alpha \;\sigma >0\;\&\;\sigma \neq 0\right]$:

(136) $$\begin{array}{c} \nu_{112}\left(x,y,z,t\right)=\frac{a_{0}\left(p+q\right)+6\left(\beta +\sqrt{\beta^{2}-4\alpha \sigma }\tanh\left(\frac{1}{2}\sqrt{\beta^{2}-4\alpha \sigma }\left(\frac{t^{\alpha_{1}}\left(4\alpha \sigma -\beta^{2}+s\right)}{r\alpha_{1}}+x+y+z\right)\right)\right)}{p+q}, \end{array}$$

(137) $$\begin{array}{c} \nu_{113}\left(x,y,z,t\right)=\frac{a_{0}\left(p+q\right)+6\left(\beta +\sqrt{\beta^{2}-4\alpha \sigma }\coth\left(\frac{1}{2}\sqrt{\beta^{2}-4\alpha \sigma }\left(\frac{t^{\alpha_{1}}\left(4\alpha \sigma -\beta^{2}+s\right)}{r\alpha_{1}}+x+y+z\right)\right)\right)}{p+q}. \end{array}$$

When $\left[\beta^{2}+4\;\alpha^{2}<0\;\&\;\alpha =-\sigma \;\&\;\sigma \neq 0\right]$:

(138) $$\begin{array}{c} \nu_{114}\left(x,y,z,t\right)=\frac{6\alpha \beta +\alpha a_{0}\left(p+q\right)-6\alpha \sqrt{-4\alpha^{2}-\beta^{2}}\tan\left(\frac{1}{2}\sqrt{-4\alpha^{2}-\beta^{2}}\left(\frac{t^{\alpha_{1}}\left(-4\alpha^{2}-\beta^{2}+s\right)}{r\alpha_{1}}+x+y+z\right)\right)}{\alpha (p+q)}, \end{array}$$

(139) $$\begin{array}{c} \nu_{115}\left(x,y,z,t\right)=\frac{6\alpha \beta +\alpha a_{0}\left(p+q\right)-6\alpha \sqrt{-4\alpha^{2}-\beta^{2}}\cot\left(\frac{1}{2}\sqrt{-4\alpha^{2}-\beta^{2}}\left(\frac{t^{\alpha_{1}}\left(-4\alpha^{2}-\beta^{2}+s\right)}{r\alpha_{1}}+x+y+z\right)\right)}{\alpha (p+q)}. \end{array}$$

When $\left[\beta^{2}+4\;\alpha^{2}>0\;\&\;\alpha =-\sigma \;\&\;\sigma \neq 0\right]$:

(140) $$\begin{array}{c} \nu_{116}\left(x,y,z,t\right)=\frac{\alpha a_{0}\left(p+q\right)+6\alpha \left(\beta +\sqrt{4\alpha^{2}+\beta^{2}}\tanh\left(\frac{1}{2}\sqrt{4\alpha^{2}+\beta^{2}}\left(\frac{t^{\alpha_{1}}\left(-4\alpha^{2}-\beta^{2}+s\right)}{r\alpha_{1}}+x+y+z\right)\right)\right)}{\alpha (p+q)}, \end{array}$$

(141) $$\begin{array}{c} \nu_{117}\left(x,y,z,t\right)=\frac{\alpha a_{0}\left(p+q\right)+6\alpha \left(\beta +\sqrt{4\alpha^{2}+\beta^{2}}\coth\left(\frac{1}{2}\sqrt{4\alpha^{2}+\beta^{2}}\left(\frac{t^{\alpha_{1}}\left(-4\alpha^{2}-\beta^{2}+s\right)}{r\alpha_{1}}+x+y+z\right)\right)\right)}{\alpha (p+q)}. \end{array}$$

When $\left[\beta^{2}-4\;\alpha^{2}<0\;\&\;\alpha =\sigma \;\&\;\sigma \neq 0\right]$:

(142) $$\begin{array}{c} \nu_{118}\left(x,y,z,t\right)=\frac{\alpha a_{0}\left(p+q\right)+6\alpha \left(\beta -\sqrt{4\alpha^{2}-\beta^{2}}\tan\left(\frac{1}{2}\sqrt{4\alpha^{2}-\beta^{2}}\left(\frac{t^{\alpha_{1}}\left(4\alpha^{2}-\beta^{2}+s\right)}{r\alpha_{1}}+x+y+z\right)\right)\right)}{\alpha (p+q)}, \end{array}$$

(143) $$\begin{array}{c} \nu_{119}\left(x,y,z,t\right)=\frac{\alpha a_{0}\left(p+q\right)+6\alpha \left(\beta -\sqrt{4\alpha^{2}-\beta^{2}}\cot\left(\frac{1}{2}\sqrt{4\alpha^{2}-\beta^{2}}\left(\frac{t^{\alpha_{1}}\left(4\alpha^{2}-\beta^{2}+s\right)}{r\alpha_{1}}+x+y+z\right)\right)\right)}{\alpha (p+q)}. \end{array}$$

When $\left[\beta^{2}-4\;\alpha^{2}>0\;\&\;\alpha =\sigma \;\&\;\sigma \neq 0\right]$:

(144) $$\begin{array}{c} \nu_{120}\left(x,y,z,t\right)=\frac{\alpha a_{0}\left(p+q\right)+6\alpha \left(\beta +\sqrt{\beta^{2}-4\alpha^{2}}\tanh\left(\frac{1}{2}\sqrt{\beta^{2}-4\alpha^{2}}\left(\frac{t^{\alpha_{1}}\left(4\alpha^{2}-\beta^{2}+s\right)}{r\alpha_{1}}+x+y+z\right)\right)\right)}{\alpha (p+q)}, \end{array}$$

(145) $$\begin{array}{c} \nu_{121}\left(x,y,z,t\right)=\frac{\alpha a_{0}\left(p+q\right)+6\alpha \left(\beta +\sqrt{\beta^{2}-4\alpha^{2}}\coth\left(\frac{1}{2}\sqrt{\beta^{2}-4\alpha^{2}}\left(\frac{t^{\alpha_{1}}\left(4\alpha^{2}-\beta^{2}+s\right)}{r\alpha_{1}}+x+y+z\right)\right)\right)}{\alpha (p+q)}. \end{array}$$

When [ασ<0&α≠0&β=0]:

(146) $$\begin{array}{c} \nu_{122}\left(x,y,z,t\right)=a_{0}-\frac{12\sqrt{\alpha \sigma }\tan\left(\sqrt{\alpha \sigma }\left(\frac{t^{\alpha_{1}}\left(4\alpha \sigma +s\right)}{r\alpha_{1}}+x+y+z\right)\right)}{p+q}, \end{array}$$

(147) $$\begin{array}{c} \nu_{123}\left(x,y,z,t\right)=a_{0}+\frac{12\sqrt{\alpha \sigma }\cot\left(\sqrt{\alpha \sigma }\left(\frac{t^{\alpha_{1}}\left(4\alpha \sigma +s\right)}{r\alpha_{1}}+x+y+z\right)\right)}{p+q}. \end{array}$$

When [ασ>0&α≠0&β=0]:

(148) $$\begin{array}{c} \nu_{124}\left(x,y,z,t\right)=a_{0}+\frac{12\sqrt{-\alpha \sigma }\tanh\left(\sqrt{-\alpha \sigma }\left(\frac{t^{\alpha_{1}}\left(4\alpha \sigma +s\right)}{r\alpha_{1}}+x+y+z\right)\right)}{p+q}, \end{array}$$

(149) $$\begin{array}{c} \nu_{125}\left(x,y,z,t\right)=a_{0}+\frac{12\sqrt{-\alpha \sigma }\coth\left(\sqrt{-\alpha \sigma }\left(\frac{t^{\alpha_{1}}\left(4\alpha \sigma +s\right)}{r\alpha_{1}}+x+y+z\right)\right)}{p+q}. \end{array}$$

When [β=0&α=−σ]:

(150) $$\begin{array}{c} \nu_{126}\left(x,y,z,t\right)=a_{0}+\frac{12\alpha \coth(\alpha \left(\frac{\left(s-4\alpha^{2}\right)t^{\alpha_{1}}}{r\alpha_{1}}+x+y+z\right))}{p+q}. \end{array}$$

When [β=σ=κ&α=0]:

(151) $$\begin{array}{c} \nu_{127}\left(x,y,z,t\right)=a_{0}+\frac{12\alpha \coth(\alpha \left(\frac{\left(s-4\alpha^{2}\right)t^{\alpha_{1}}}{r\alpha_{1}}+x+y+z\right))}{p+q}. \end{array}$$

When [α=0]:

(152) $$\begin{array}{c} \nu_{128}\left(x,y,z,t\right)=a_{0}+\frac{12\beta (\frac{2}{\sigma e^{\beta (\frac{\left(s-\beta^{2}\right)t^{\alpha_{1}}}{r\alpha_{1}}+x+y+z)}-2}+1)}{p+q}. \end{array}$$

When [β=α=0]:

(153) $$\begin{array}{c} \nu_{129}\left(x,y,z,t\right)=a_{0}+\frac{12r\alpha_{1}}{\left(p+q\right)(r\alpha_{1}\left(x+y+z\right)+st^{\alpha_{1}})}. \end{array}$$

When [β=0&α=σ]:

(154) $$\begin{array}{c} \nu_{130}\left(x,y,z,t\right)=a_{0}-\frac{12\alpha \tan(C+\alpha \left(\frac{\left(4\alpha^{2}+s\right)t^{\alpha_{1}}}{r\alpha_{1}}+x+y+z\right))}{p+q}. \end{array}$$

When $\left[\beta =\sqrt{4\;\alpha \;\sigma }\right]$:

(155) $$\begin{array}{c} \nu_{131}\left(x,y,z,t\right)=a_{0}+\frac{6(2\sqrt{\alpha \sigma }+\frac{2r\alpha_{1}}{r\alpha_{1}\left(x+y+z\right)+st^{\alpha_{1}}})}{p+q}. \end{array}$$

## 3. Physical Interpretation of Solution

This section discusses and interprets some of the obtained solutions under the suitable choice of the parameters values as shown in Table 1. Generally, all of these waves are considered as traveling from right to left.

## 4. Discussion

This section investigates the relation between all above-mentioned methods with a modified auxiliary equation method (modified Khater method). We show the similarities and differences between them and the result of this discussion is shown in Table 2.

According to this discussion, we can conclude that the modified auxiliary equation method (modfied Khater method) covers the first seven methods that are used in this research and it is more general than them.

## 5. Conclusions

This paper studied the performance of conformable fractional derivative on the time fractional Jimbo–Miwa equation. Moreover, nine analytical and modified auxiliary equation methods (modified Khater method) were applied to this model for getting various explicit wave solutions of the fractional JM model. The solutions obtained were discussed and represented under the suitable choice of the parameters to show the physical properties of each one of them. In addition, we studied each one of the used methods and its relation with the modified auxiliary equation method. Our discussion shows the superiority of the modified auxiliary equation method (modified Khater method) on some of these methods such that it covers almost all solutions that are obtained by these methods.

## Figures and Tables

**Table 1:** Physical interpretation of represented solutions where S,A,andW represent shape, amplitude, and wavelength, respectively.

Fig. Nu.	S	A	W	Parameters Value
$\nu_{1}\left(x,t\right)$	Periodic kink	7.4	6	$\left[a_{0}=9,\;\alpha_{1}=0.5,\;\lambda =3,\;\mu =2,\;p=8,\;q=7,\;r=10,\;s=5,\;y=1,\;z=4,\;\vartheta =6\right]$
$\nu_{12}\left(x,t\right)$	Singular	15	0.5	$\left[r=4,\;a_{0}=9,\;\alpha_{1}=0.5,\;\mu =2,\;p=8,\;q=7,\;s=5,\;y=1,\;z=10\right]$
$\nu_{18}\left(x,t\right)$	Singular kink	15	7	$\left[a_{0}=9,\;\alpha_{1}=0.5,\;C_{1}=11,\;C_{2}=12,\;\mu =-4,\;p=8,\;q=7,\;r=10,\;s=5,\;y=1,\;z=2\right]$
$\nu_{27}\left(x,t\right)$	Singular	20	1.9	$\left[a_{0}=9,\;\alpha_{1}=0.5,\;d=4,\;p=8,\;q=7,\;r=10,\;s=5,\;y=1,\;z=2\right]$
$\nu_{33}\left(x,t\right)$	Periodic kink	1.2	0.7	$\left[\alpha_{1}=0.5,\;a=4,\;b=-2,\;y=1,\;z=2\right]$
$\nu_{39}\left(x,t\right)$	periodic kink	53	7	$\left\{\alpha_{1}=0.5,\;a_{0}=5,\;b=-2,\;\lambda =4,\;\mu =-1,\;p=-2,\;q=3,\;r=-3,\;z=2,\;\vartheta =3\right\}$
$\nu_{52}\left(x,t\right)$	Kink	10	6	$\left[\alpha_{1}=0.5,\;a_{0}=5,\;A=2,\;B=3,\;\Delta =1,\;m=1,\;r=-3,\;s=6,\;y=1,\;z=2\right]$
$\nu_{79}\left(x,t\right)$	periodic anti-kink	15	6	$\left[\alpha_{1}=0.5,\;A_{0}=3,\;r=-1,\;s=5,\;y=1,\;z=2\right]$
$\nu_{82}\left(x,t\right)$	periodic kink	0.0006	6	$\left[\alpha_{1}=0.5,\;c=1,\;\mu =5,\;p=3,\;q=4,\;r=2,\;s=6,\;y=1,\;z=2\right]$
$\nu_{90}\left(x,t\right)$	periodic kink	0.1	6.5	$\left[a_{0}=7,\;\alpha =1,\;\beta =3,\;c=1,\;p=3,\;q=4,\;r=5,\;s=6,\;\sigma =2,\;y=1,\;z=2,\;\alpha_{1}=0.5\right]$

**Table 2:** Discussion of the relations between the modified auxiliary equation and above-mentioned methods.

Method	Conditions	Similar
Exp (−ϕ(Θ))-expansion method	[f(Θ)=ϕ(Θ),e=K,σ=μ,β=λ,α=1]	*√*
Improved F-expansion method	$\left[K^{f(\Theta )}=\mu +\phi \left(\Theta \right),\;\sigma =1,\;\beta =-2\;\mu ,\;\alpha =\mu^{2}+r\right]$	*√*
Extended $\left(\frac{G^{\prime }}{G}\right)$-expansion method	$\left[K^{f(\Theta )}=\frac{G^{\prime }\left(\Theta \right)}{G(\Theta )},\;\sigma =-\mu ,\;\beta =0,\;\alpha =-1\right]$	*√*
Extended tanh- function method	$\left[K^{f(\Theta )}=\phi \left(\Theta \right),\;\sigma =1,\;\beta =0,\;\alpha =d\right]$	*√*
Simplest equation method	$\left[K^{f(\Theta )}=f^{*}\left(\Theta \right),\;\sigma =c_{2},\;\beta =c_{1},\;\alpha =0\right]$	*√*
Extended simplest equation method	$\left[K^{f(\Theta )}=f^{*}\left(\Theta \right),\;\sigma =\mu ,\;\beta =\lambda ,\;\alpha =\alpha^{*}\right]$	*√*
Generalized Riccati expansion method	$\left[K^{f(\Theta )}=\phi \left(\Theta \right),\;\sigma =m,\;\beta =B,\;\alpha =A\right]$	*√*
Generalized Sinh–Gordon expansion method	[⋯⋯⋯⋯⋯⋯⋯⋯⋯⋯⋯]	*x*
Riccati–Bernoulli Sub-ODE method	[⋯⋯⋯⋯⋯⋯⋯⋯⋯⋯⋯]	*x*
